# Scalable Ni-Based Diffusion Synthesis of Highly Graphitic Nanointerlaced and Photopatternable Material with Fast Charge Transfer Kinetics

**DOI:** 10.1021/acs.langmuir.5c06421

**Published:** 2026-05-21

**Authors:** Carina Chávez-Granados, Pedro Roquero, Oscar Pilloni, Margarita Rivera, Marc J. Madou, Laura Oropeza-Ramos

**Affiliations:** a Facultad de Ingeniería, 7180 Universidad Nacional Autónoma de México, Ciudad Universitaria, Ciudad de México 04510, México; b Departamento de Ingeniería Química, Facultad de Química, Universidad Nacional Autónoma de México, Ciudad Universitaria, Ciudad de México 04510, México; c Instituto de Ingeniería, Universidad Nacional Autónoma de México, Ciudad Universitaria, Ciudad de México 04510, México; d Instituto de Física, Universidad Nacional Autónoma de México, Ciudad Universitaria, Ciudad de México, 04510, México; e Tecnológico de Monterrey, School of Engineering and Sciences, Ave. Eugenio Garza Sada 2501 Sur, Monterrey, N.L. 64849, México

## Abstract

Graphitic and nanostructured carbon materials exhibit exceptional properties, including high electrical conductivity and chemical stability, yet their integration into microdevices remains challenging due to costly, low-yield production methods. Carbon-MEMS (C-MEMS) technology offers a scalable alternative for fabricating carbon microdevices, though it typically yields amorphous/glassy carbon with limited conductivity. Here, we bridge this gap with a scalable method for synthesizing highly conductive graphitic micropatterns. Our approach combines electrospinning of a photosensitive SU-8/MWCNT composite with pyrolysis and catalytic graphitization using a Ni film. Raman spectroscopy confirms increased graphitization (*I*_D_/*I*_G_ = 0.3 vs 0.92 for pyrolytic carbon), and HRTEM analysis reveals numerous graphitic domains with parallel lattices exhibiting an interlayer distance of 3.46–3.59 Å. These observations are consistent with the substantial decrease in the *I*_D_/*I*_G_ ratio and the corresponding increase in crystallite size (*L*_a_) estimated by Raman spectroscopy, confirming the evolution of disordered carbon toward a long-range preferred orientation. This structural transformation is promoted by the Ni-based diffusion mechanism specific to our porous architecture. The process results in carbon nanofiber mats with enhanced electrical conductivity (CI_95%_ [615.0, 824.2] S/m), a significant improvement over pyrolytic mats from the same composite (CI_95%_ [4638.1, 5625.9] S/m). Electrochemically, the material exhibits superior charge-transfer kinetics (*k*° = 0.064 cm/s), outperforming some conventional carbons. Our investigations reveal that the inclusion of MWCNTs reduces porosity, thereby increasing conductivity by 3 orders of magnitude, which allows for the surface area and electron-transfer tuning. Furthermore, our Ni-based diffusion synthesis enables the direct integration of micropatterned, nanointerlaced graphitic devices into silicon substrates, eliminating transfer steps or the need for mechanical treatment while enabling complex geometries. In summary, this work provides a large-scalable route for the next generation of high-performance graphitic devices with broad applicability in miniaturized sensors and energy systems.

## Graphical abstract

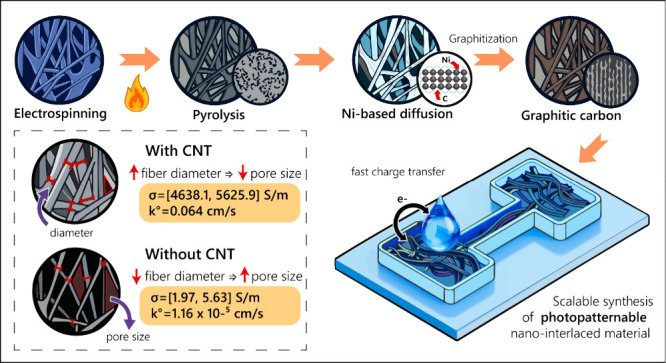

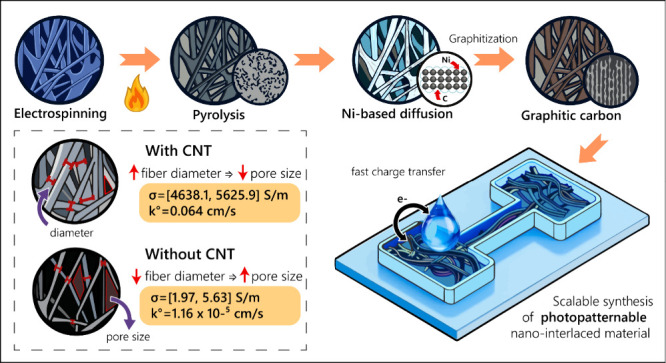

## Introduction

Large-scale microdevice fabrication nowadays is feasible due to silicon’s well-known properties. Silicon is one of the most transformative materials for humankind and has undoubtedly revolutionized how people interact through electronic devices. On the other hand, carbon is a key element of life due to its extraordinary capacity to form stable and complex bonds, allowing it to create the backbone of core molecules of biology and of organic chemistry. Carbon’s versatility knows no bounds, making it a material of high scientific and technological relevance with unique properties (chemical, physical, and electronic) offered by its diverse allotropic forms. Carbon allotropes exhibit common characteristics, including high mechanical strength, good thermal conductivity, high electrochemical stability, chemical inertness, and biocompatibility, among others,^1^ which positions them as ideal materials for devices where conventional silicon technology has limitations.

Carbon’s interesting properties depend on the type of hybridization formed by the carbon atoms in the material’s microstructure.^2^ Since carbon allotropes exhibit a wide range of hybridizations, a wide range of conductivities is obtained.^3^ When sp^2^ hybridization dominates, one electron per carbon atom remains unpaired, and the presence of π states within the sp^2^ bonds governs the carbon conductivity.^4^

Significant effort has been devoted to ordering carbon atoms to achieve outstanding properties of materials like graphite and graphene. This process, known as graphitization, is the gradual conversion of noncrystalline carbon into graphite via high-temperature oxygen-free heat treatment (pyrolysis). In 1951, Rosalind Franklin stated that “the structure of pyrolytic carbons depends not only on the preparation temperature but also on the nature of the starting material”.^5^ She classified polymeric and organic precursors into two groups: graphitizing carbons, which exhibit a homogeneous graphitic structure, and nongraphitizing carbons, which maintain their disordered structure. Harris^6^ later explained that these nongraphitizable carbons formed particle-like fullerenes, which prevented the formation of crystalline graphite. From that moment on, the interest in investigating the structure of pyrolytic carbons increased, and the advent of transmission electron microscopy (TEM) led to the discovery of diverse graphitic nanostructures, including nano-onions and bucky onions, nanoribbons, nanofibers, and fullerenes, generated through industrial carbonization, arc-discharge, and laser irradiation. In 1968, Heidenreich observed concentric polyhedral graphitic layers during the graphitization of carbon black (CB).^7^ Oberlin reported similar structures in graphitizing semicoke and anthracite, describing how pore walls transform into concentric layers of “partially graphitized carbon”.^8^ Ugarte later coined terms like “nano-onions” and “bucky onions” for these shapes, produced by electron beam irradiation.^9,10^ Earlier breakthroughs, such as the discovery of buckminsterfullerene (C_60_) by Kroto et al.^11^ and the subsequent identification of carbon nanotubes (CNTs) by Iijima,^12^ marked key milestones in nanomaterials research because they retain the exceptional physicochemical properties of traditional carbon allotropes (graphene and graphite).

Despite this progress in identifying new carbon nanomaterials, manufacturing carbon devices on a large scale remains challenging. For instance, a pristine graphene produced by CVD requires a film transfer to Si substrates for electronic applications.^13^ This step increases defects and film contamination, adversely affecting electron transport and, therefore, overall device performance. While direct synthesis of graphene on dielectric substrates has gained interest, significant issues persist. Techniques for epitaxial growth on materials such as silicon oxides/nitrides, SiC, and sapphire often result in low-growth rates, uncontrolled layer thickness, and uneven film quality.^13,14^ Jiang et al. developed an LPCVD-based method to synthesize graphene directly on dielectric wafers using ethanol as a precursor. They patterned the graphene via UV-lithography and O_2_-RIE etching.^15^ Although this approach enables batch production and avoids film transfer steps, the RIE process itself, with its slow etching rates and specific equipment demands, limits its scalability.^16^

In a related approach, synthesis of bilayer graphene by annealing Ni-coated polymers (PMMA, PS, ABS) in an inert atmosphere was reported.^17,18^ This process is governed by Ni’s low carbon solubility, which allows carbon to dissolve into the face-centered cubic (FCC) lattice, diffuse, and, after cooling, segregate into an ordered graphitic structure.^19^

Numerous articles have reported that the synthesis of graphene on Ni is a complex, multistage process driven by the interstitial diffusion of carbon and the temperature-dependent solubility of carbon in Ni. This three-stage process is depicted schematically in Figure 1. It is initiated by the catalytic dissociation of carbon and its subsequent interstitial diffusion (stage 1), which is facilitated by the high diffusion rate of carbon and the high carbon solubility of Ni (0.9 atom % at 900 °C).^19–21^ During the high-temperature stage (stage 2), segregation emerges as a thermodynamic equilibrium phenomenon driven by the minimization of the Gibbs free energy of the system, since the surface energy of graphene (46.7 mJ/m^2^) is much lower than that of Ni (1900 mJ/m^2^).^22^ The nucleation of six-membered carbon rings creates a carbon sink with lower chemical potential, reversing the diffusion flux and driving carbon toward the surface.^23^ Finally, precipitation occurs during cooling (stage 3). The drastic drop in carbon solubility in Ni causes the expulsion of excess dissolved carbon in an ordered form.

**Figure 1:**
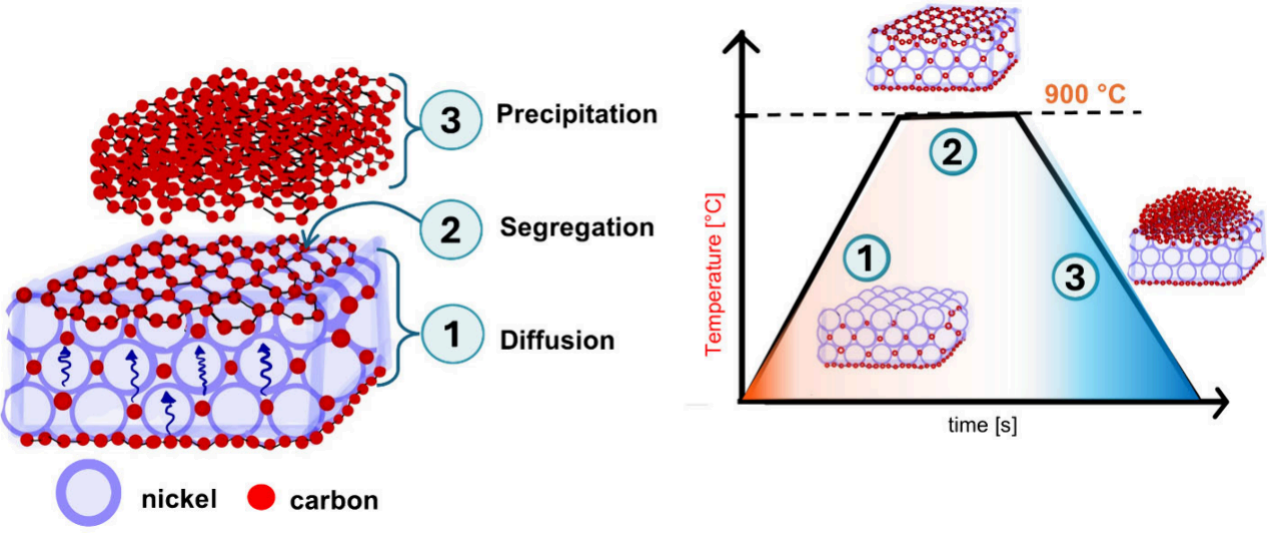
Schematic of the three-stage growth mechanism of graphene during a temperature–time profile: (1) diffusion of carbon into the Ni; (2) segregation of carbon atoms toward the surface at high temperature; (3) precipitation of ordered graphitic layers during cooling.

In 2021, our group harnessed this diffusion and segregation mechanism to order pyrolytic carbon derived from thin films of SU-8 photoresist via carbon-MEMS (C-MEMS), a method known for using polymeric precursors to create complex carbon shapes.^24^ Subsequently, Ni-catalyzed annealing transformed these C-MEMS structures into graphitic micropatterns, a novel methodology bridging a fundamental materials process with microtechnology industrial applications.^25^

On the other hand, the use of composites in C-MEMS technology has also garnered attention to enhance the properties of polymeric precursors, and carbon nanotubes (CNTs) are a very common reinforcement to improve mainly mechanical and electrical properties,^26^ even at relatively low concentrations of CNTs.^27^ The electrospinning of these composites allows the production of mats with highly aligned fibers.^28^ In 2017, our group participated in the graphitization of polyacrylonitrile (PAN) from a composite with PAN and multiwalled carbon nanotubes (MWCNTs). Through an electrospinning process, the orientation of the molecular chains in polymer precursor is promoted due to both the dielectrophoresis phenomenon caused by the difference in electrodynamic forces between CNTs and polymer and to the mechanical treatment.^29^ However, PAN is not photosensitive.

The work presented here seeks to use the electrodynamic effect of depositing a composite of SU-8 with MWCNT by electrospinning, obtaining carbon nanofibers through a vacuum-atmosphere pyrolysis process, followed by diffusion of carbon atoms through a thin film of Ni, to synthesize a novel, scalable, highly ordered material with improved electrical/electrochemical properties for the development of C-MEMS devices. Our synthesis method has the advantage of starting from a photosensitive precursor widely used in the microfabrication industry, which allows both planar and extruded microstructures to be directly integrated into the substrate, and it is compatible with existing silicon technology processes. The resulting carbon has a graphitic structure, with improved conductivity compared to SU-8-derived pyrolytic carbon and a high surface area due to its nanointerlaced nature, which may be beneficial for a broad range of electrochemical applications. Furthermore, the use of electrospinning enables dimensional control. These advantages can potentially resolve the challenges of integrating nanomaterials like graphene and carbon nanotubes, offering a novel path for next-generation carbon devices.

## Materials and Methods

### Precursors Preparation for Electrospinning

#### SU-8

A solution of 70 wt % SU-8 2100 (Microchem), 15 mg of poly-ethylene oxide (PEO, Sigma-Aldrich), and 25 mg of tetrabutylammonium tetrafluoroborate (Sigma-Aldrich) was dissolved in 30 wt % cyclopentanone (Sigma-Aldrich) by magnetic stirring at 50 °C for 24 h.

#### SU8+MWCNT

SU-8 from the previous preparation and 1 wt % MWCNT (Sigma-Aldrich, >98% carbon basis, with a diameter of 6 to 13 nm and a length of 2.5–20 μm) were dissolved in 30 wt % cyclopentanone (Sigma-Aldrich) by magnetic stirring at 50 °C for 24 h.

### Ni-Based Diffusion Synthesis

The synthesis process is graphically illustrated in Figure 2, and each stage is described in a progressive order as follows.

**Figure 2:**
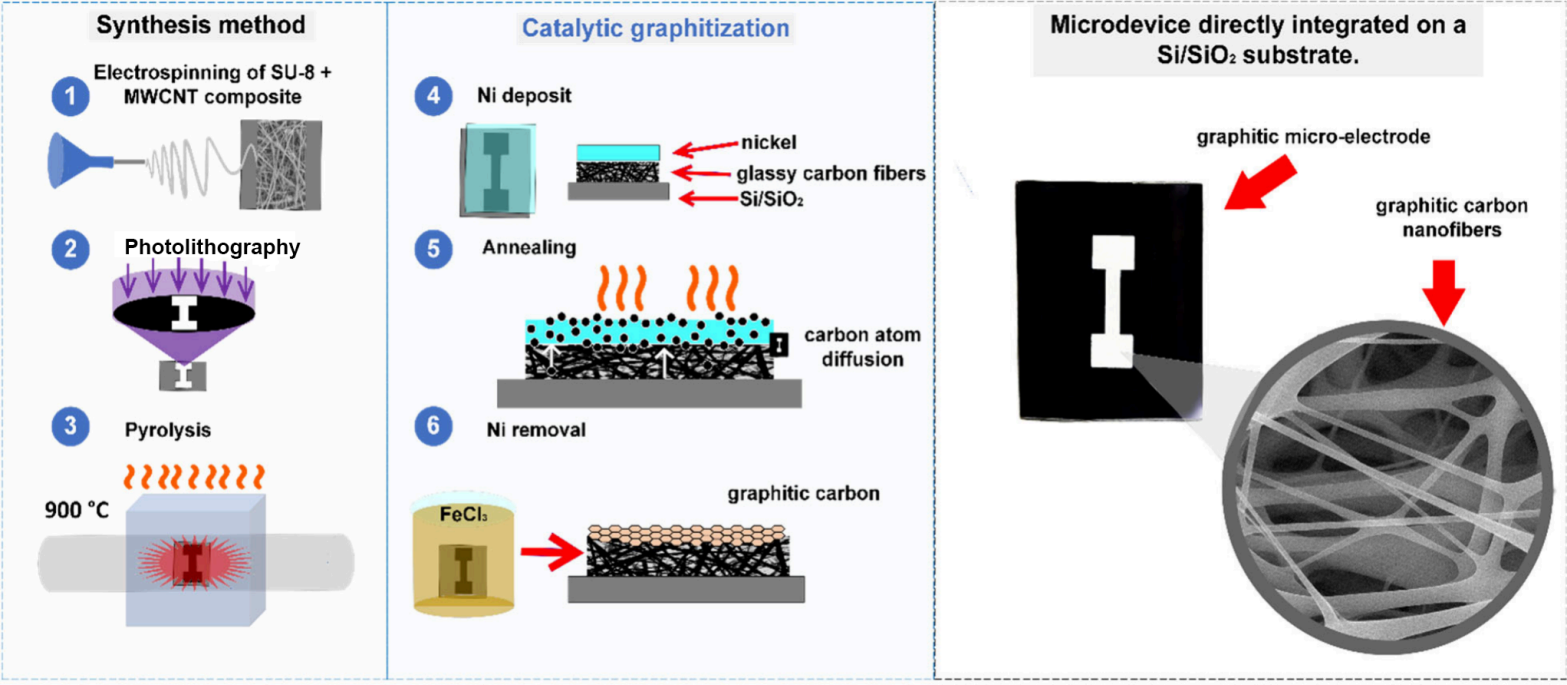
Schematic of the synthesis route: steps 1–3, composite electrospinning, photopatterning, and pyrolysis at 900 °C. During steps 4–6 catalytic graphitization process occurs, which structurally orders the carbon material formed during the initial pyrolysis steps. A microdevice with nanointerlaced architecture composed of graphitic carbon nanofibers is shown.

#### Fiber Deposition by Electrospinning

For the electrospinning process, the SU-8 MWCNT composite was ultrasonicated at 11 000 rpm for 10 min and ejected with a 3 mL KD Scientific syringe pump at a flow rate of 2 μL/h. A high voltage (10 kV, Stanford Research Systems +20 kV supply) was applied between a 21-gauge ejector needle and a rotating collector, separated by 8 cm. Nanofibers were deposited onto silicon substrates coated with a 90 nm layer of silicon dioxide (Graphene Supermarket, resistivity of 0.001–0.005 Ω·cm) for 6 h to form nanointerlaced films (nanofiber mats) of around 40 μm in thickness, using a custom-built electrospinning previously reported.^30^ While samples in this study were limited to 1–2 cm^2^ due to laboratory equipment constraints, the electrospinning of SU-8/MWCNT composites is fundamentally scalable to large-area deposition using industrial multinozzle systems.

#### Photolithographic Patterning

Microelectrodes were patterned onto the nanointerlaced films using maskless photolithography system (SF100 XCEL Intelligent Micro Patterning) under the following conditions: exposure time of 280 s, postbake at 95 °C for 5 min, soak in SU-8 developer for 5 min with moderate agitation, dried with N_2_, and hard bake at a rate increment of 10 °C/min until reaching 190 °C for 1 h. The cooling was performed at a rate of 10 °C/min until room temperature was reached (Figure S1). The use of photosensitive SU-8 as a precursor enables high-throughput parallel patterning. Unlike serial lithography methods, this approach allows for the simultaneous fabrication of complex microstructures across entire wafers, aligning with standard semiconductor manufacturing throughput requirements.

#### Pyrolysis

The pyrolysis was performed in a CVD oven (OTF-1200X-80-SL, MTI Corporation) at 3.0–3.9 mTorr vacuum, following the temperature ramp reported by Vaca et al.^25^

#### Ni Deposition and Diffusion: Catalytic Graphitization

A 100 nm Ni film was deposited on top of the carbon nanofibers film by thermal evaporation at a current of 30 A, 10 mTorr of pressure, and at a rate of 1.8 Å/s. Given the influence of catalyst thickness on the structural ordering of volumetric interfaces remains underexplored in the literature, this parameter was selected as the initial variable of investigation. The chosen thickness was reproducibly achieved using a custom-built (noncommercial) thermal evaporation system, which ensured uniform film deposition and adequate adhesion, thereby preventing delamination during subsequent thermal processing.

This was followed by annealing at 900 °C for 20 min with a cooling rate of 10 °C/min, under a pressure of 2.9 to 3.5 mTorr. Finally, Ni was removed by immersing the samples in a FeCl_3_ solution for 5 min, followed by rinsing with deionized water and isopropyl alcohol. To evaluate the effectiveness of the Ni removal we realized SEM-EDS for large areas of the top layer as well as for the cross section, and HRTEM-EDS analysis to differentiate Ni residual nanoparticles from the graphitic matrix, confirming in all cases that at the final synthesis step Ni concentrations are below 0.5 atom % (Tables S7–S10 and Figures S8–S10).

### Carbon Characterization Methods

Given the high surface roughness and porosity inherent to the electrospun fibrous mats, Raman spectra were acquired through manual point-selection to optimize focal accuracy and signal-to-noise ratio. Raman spectroscopy was performed at two distinct steps of the carbon material synthesis using a Thermo Scientific Raman microscope DXR equipped with a 532 nm laser excitation source: (i) after pyrolysis (step 3 in Figure 2) and (ii) following the Ni removal process (step 6 in Figure 2). Data were collected on 8 samples at each processing step. For the pyrolytic material (step 3), one measurement per sample was acquired, resulting in a total of 8 spectra. To ensure representative sampling of the material’s heterogeneity after Ni removal (step 6), two points per sample, one at the center and one at the corner, were examined, yielding 16 spectra for the graphitic material.

Electrical conductivity was measured using the four-point probe method after steps 3 and 6 of the synthesis using a Keithley 2400 SourceMeter and a Jandel probe head. Conductivity measurements were performed at two locations per sample on 6 graphitized (step 6) and 4 pyrolytic (step 3) samples, with only reproducible *I*–*V* curves included in the final analysis.

A JEOL JEM-ARM200F transmission electron microscopy TEM instrument operated at 200 kV was used to take high-resolution HRTEM images of material samples transferred onto a CF 300 CU-50 (carbon film 300 mesh copper) grid.

The electrochemical behavior of the material after steps 3 and 6 was characterized by cyclic voltammetry (CV) using potassium ferri/ferrocyanide as the redox couple. The electrochemical cell was composed of a solution of 10 mM potassium ferricyanide K_3_[Fe­(CN)_6_] (Sigma-Aldrich) in 2 M potassium chloride (Sigma-Aldrich) using a traditional 3-electrode configuration with a saturated calomel SCE reference electrode and a graphite counter electrode. A P5T050 analytical voltammetry instrument of Voltalab was used.

## Results and Discussion

### Raman Spectroscopy and Conductivity Characterization

In Figure 3a, we show the results of Raman spectroscopy for both pyrolyzed and graphitic carbon (after Ni-based diffusion synthesis) electrodes, from which the relationship between the intensities of the D and G bands (*I*_D_/*I*_G_) was calculated as an indicator for the degree of structural disorder of the material. For SU-8+MWCNT pyrolytic (without Ni diffusion) two main bands are observed: band D associated with structural defects in the graphitic network around 1350 cm^–1^, and band G attributed to a graphitic structure (ordered) around 1590 cm^–1^. This material showed an *I*_D_/*I*_G_ = 0.92, typical of glassy carbon (GC). After the carbon atoms diffusion process, the Raman spectrum of SU-8+MWCNT graphitic reveals three bands: D at 1338 cm^–1^, G at 1569 cm^–1^, and a 2D band characteristic of graphitic materials at 2680 cm^–1^, with a smaller D band and an average *I*_D_/*I*_G_ = 0.3. Clearly, defects have drastically decreased after the synthesis proposed here.

**Figure 3:**
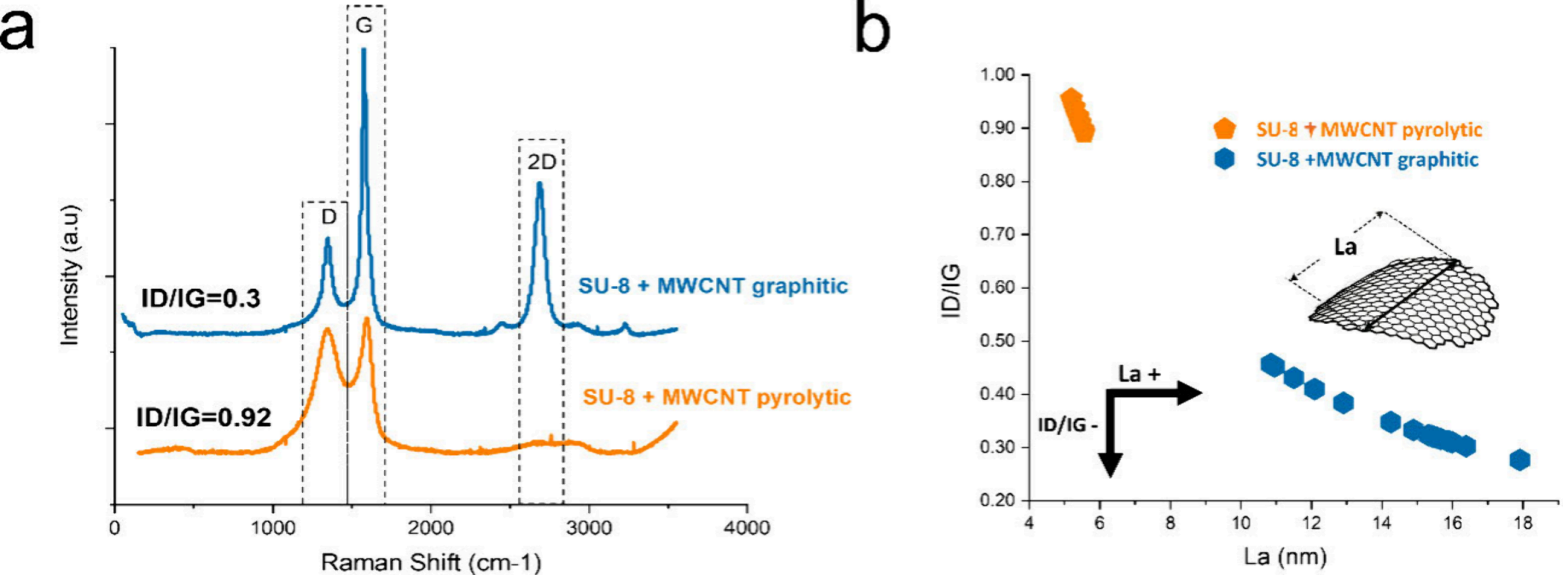
Structural evolution of SU-8+MWCNT composite analyzed by Raman spectroscopy. (a) Raman spectra of the composite after pyrolysis (step 3) and after final synthesis (step 6). (b) Relationship between the intensity ratio *I*_D_/*I*_G_ and crystallite size *L*_a_ for pyrolytic and graphitic samples. The plot is constructed from Raman data (Tables S1 and S2) using eq 3.

To further analyze this drastic dropping of the carbon material defects we evaluate the average interdefect distance *L*_a_ from its relationship with the ratio *I*_D_/*I*_G_. The latter is given by the Knight and White relationship,^31^ defined for λ = 514.5 nm as:

1 $$L_{a}=C\left(\lambda \right){\left(\frac{I_{D}}{I_{G}}\right)}^{-1};,L_{a}=4.4{\left(\frac{I_{D}}{I_{G}}\right)}^{-1}$$

Since our Raman data are obtained with a different laser wavelength, a relationship proposed by Matthews et al.^32^ was used to adjust the Knight and White relationship as follows:

2 $$C\left(\lambda \right)=C_{0}+\lambda C_{1};,\mathrm{from this}C_{0}=-12.6\mathrm{nm}\mathrm{and}C_{1}=0.033$$

Thus, for a 532 nm laser excitation we obtain the following expression:

3 $$L_{a}=\left(0.033\left(532\right)-12.6\right){\left(\frac{I_{D}}{I_{G}}\right)}^{-1};,L_{a}=4.956{\left(\frac{I_{D}}{I_{G}}\right)}^{-1}$$

Using the *I*_D_/*I*_G_ Raman ratios for both pyrolytic and graphitic (Tables S1 and S2), we constructed the plots illustrated in Figure 3b. These plots reveal an increase in *L*_a_ from approximately 5 nm in the pyrolytic state to values of 10–18 nm in the graphitic state as a result of our synthesis. The extension of graphitic domains aligns with Ferrari’s statement that, even for disordered graphite, *L*_a_ increases as crystalline regions grow resulting in a larger distance between defects.^33^

The results of electrical conductivity measurements show that the increased graphitization, induced by the Ni-based diffusion synthesis, resulted in a significant improvement in electrical conductivity, which rose from 719.60 ± 99.8 [S/m] in pyrolytic carbon, to 5132 ± 470.5 [S/m], after catalytic graphitization (Tables S3–S6, Figures S3–S7).

Several polymers have been used as glassy carbon precursors in the fabrication of C-MEMS electrodes, including PAN, SU-8 photoresist, polyfurfuryl alcohol, etc. In Table 1, we present the electrical conductivity data and I_D_/I_G_ values for diverse types of electrodes processed by electrospinning of typical polymeric precursors. Our nanointerlaced (or porous) carbon conductivity is one of the highest among the C-MEMS electrodes reported on, along with the PAN nanofibers subjected to mechanical compression.

**Table 1:** Electrical Conductivity Statistics Considering 95% Confidence Intervals (CI) and *I*_D_/*I*_G_ Ratio Comparison with Different Carbons

C-MEMS electrode precursor electrospun and pyrolyzed	conductivity [S/m]	*I*_D_/*I*_G_	ref
SU-8 + Ni diffusion	1.97–5.63	0.37	Current work
Poly(furfuryl alcohol)	2–15	—	^34^
PAN	150–250	1.26	^29^
PAN-CNT	450–600	1	^29^
SU-8+MWCNT	615.0–824.2	0.92	Current work
PAN-CNT tensile	700–900	0.69	^29^
PAN-CNT compressive	4900–5000	0.69	^29^
SU-8+MWCNT + Ni diffusion	4638.1–5625.9	0.3	Current work

### HRTEM Graphitic Carbon Analysis

The evolution from the precursor to the graphitic state was also observed via HRTEM. Micrographs of the composite fibers (Figure 4a), reveal an amorphous structure, in contrast to the numerous ordered and closely stacked layers of graphitic domains (Figure 4b) present at the final stage of the Ni-based diffusion synthesis. Notice that graphitic layers are aligned parallel to the fiber axis, from the edge and along the fiber surface as depicted in b_1_ and b_2_, respectively. Furthermore, the selected area electron diffraction (SAED) pattern (Figure 4c) indicates an interlayer distance (*d*_002_) of 3.46–3.59 Å, similar to the interlayer distance of graphite and MWCNT of ∼3.4 Å.^35^ Out of 56 HRTEM images analyzed, a localized presence of MWCNTs was observed in only one SU-8+MWCNT fiber (presynthesis) micrograph (Figure 4d). This scarcity is likely attributable to the low percentage of MWCNT used during the composite preparation.

**Figure 4:**
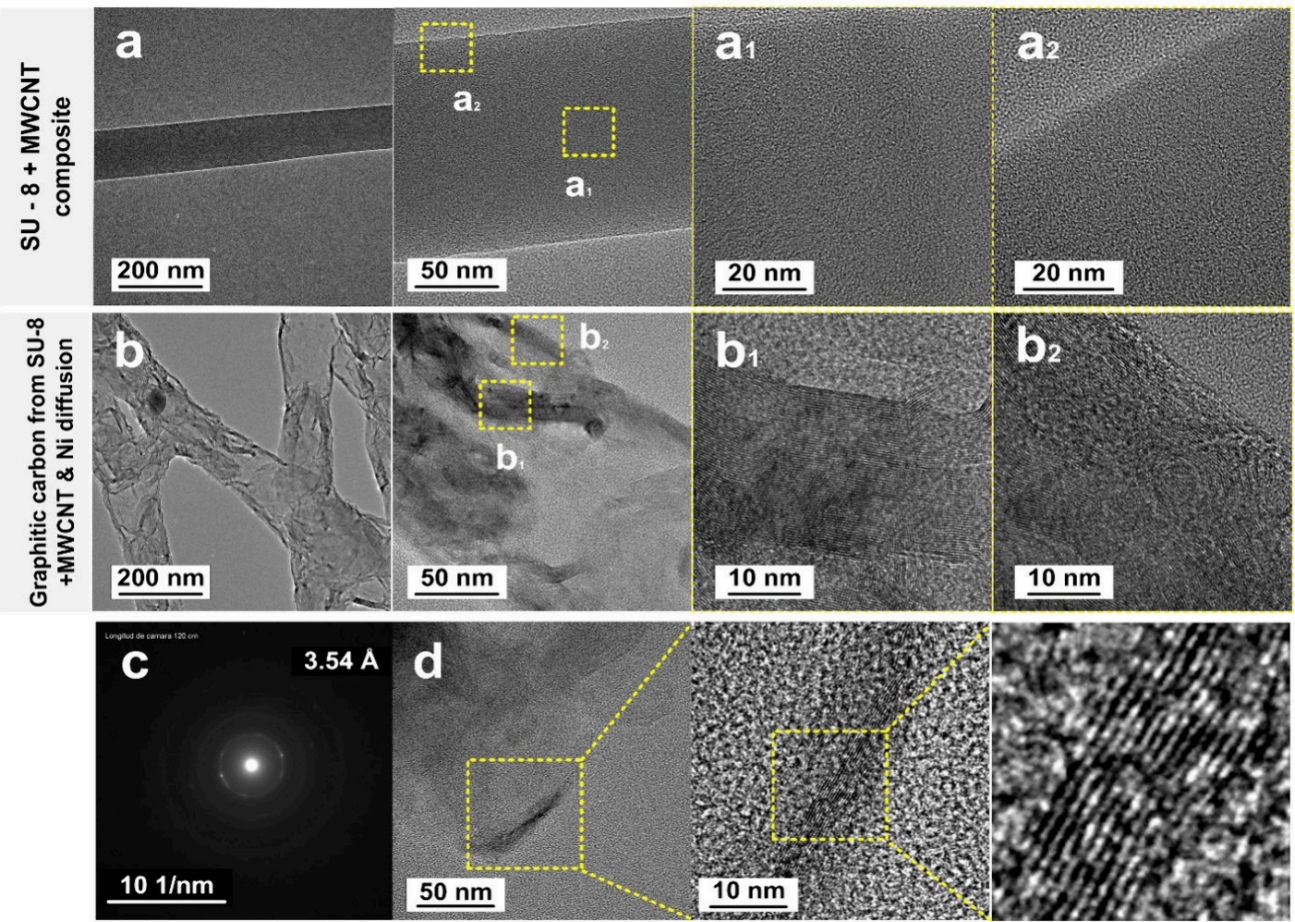
Nanofibers HRTEM micrographs of (a) composite, where a_1_ and a_2_ show the middle and the edge region, respectively. An amorphous structure is observed throughout the fiber. (b) SU-8+MWCNT graphitic carbon fiber. b_1_ and b_2_ highlight a region with aligned graphene layers, at the fiber’s edge, parallel alignment to the fiber axis is evident. (c) Selected area electron diffraction (SAED) pattern of SU-8+MWCNT graphitic and (d) amplification region of the composite nanofibers (presynthesis) where one carbon nanotube is displayed in the polymer.

We emphasize that the microscopy evidence of the ordered carbon lattices in Figure 4b,c is consistent with the drastic drop in the *I*_D_/*I*_G_ ratio and the consequent increment in crystallite size (*L*_a_) previously estimated by Raman spectroscopy. These observations confirm the evolution of disordered carbon toward a long-range preferred orientation promoted by the Ni-based mechanism specific to our porous structures.

Following the 3-stage process outlined in the introduction (Figure 1), our synthesis is then governed by the interstitial diffusion of carbon from the SU-8 pyrolytic nanofibers into the Ni catalytic film coating the porous mat. As the Ni layer functions as a sacrificial template, graphitization is confined to the fiber surfaces, where the metal has penetrated the interstitial voids. The bidirectional nature of this diffusion results in the formation of graphitic layers on both sides of the Ni film, consistent with observations reported for solid-precursor synthesis.^17,18^ The Ni lattice serves as a crystalline scaffold, promoting the development of ordered graphitic domains that remain anchored to the nanointerlaced material after the top layer is lifted off and the Ni is chemically removed (Figure S2).

Beyond the large carbon-aligned layer domains, HRTEM also reveals localized carbon nanostructures present in our material. As shown in Figure 5, pseudographitic curved shapes can be observed. Specifically, Figure 5a displays curved graphitic layers around a Ni nanoparticle (confirmed by EDS in Figure S9). This may suggest that partially graphitic layers are generated around the transition metal, adopting its contour,^36^ which are known as carbon nanocapsules,^37^ as well as bucky onions and nano-onions.^10^

**Figure 5:**
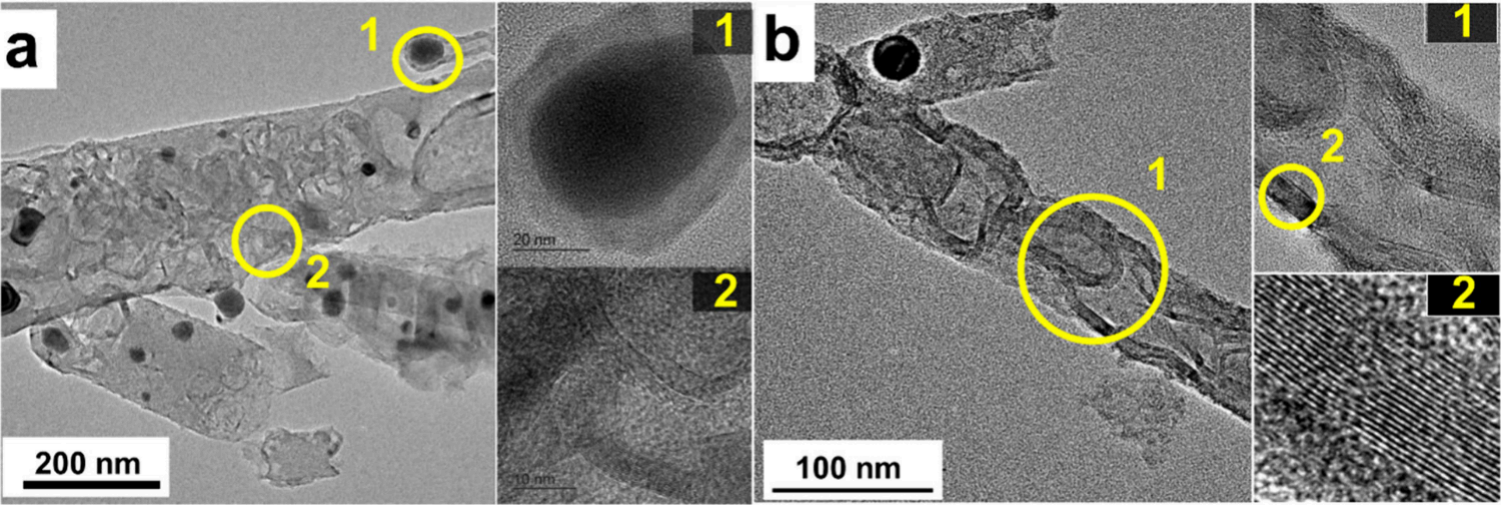
Localized carbon nanostructures: (a) partially graphitic layers wrapping the perimeter of a Ni nanoparticle (1) and concentric partially graphitic layers with polygonal shapes (2); (b) bamboo-like tubular structures.

In Figure 5b we also observed tubular structures that have been recognized as bamboo-like^38^ or chain-like fibers^39^ that have been synthesized using Cu nanoparticles^40^ and Ni nanoparticles^41^ as metal catalysts. In accordance with historical observations by Oberlin and Heidenreich, these concentric structures can be understood as semigraphitic intermediates typical of the transformation of nongraphitizing carbons.

The brief historical review provided in the Introduction, from Franklin’s foundational classification of graphitizability to Harris’s fullerene-like particles to study the transition from disordered, nongraphitizing carbons to materials with long-range, three-dimensional stacking order, is interesting for our discussion. Precursor SU-8 has been considered to resist graphitization due to the presence of fullerene-like particles reported in its GC state.^6,42^ Therefore, it is possible that fullerene-like particles in our pyrolytic SU-8 were deconstructed by the Ni catalyst, facilitating carbon atoms reorganization into the predominant graphitic domains along the fiber, in company with the observed localized nanostructures, which would be of interest for future investigations.

### Electrochemical Behavior

Cyclic voltammograms were obtained for different scan rates from 10 mV/s to 100 mV/s, with a voltage window of −0.2 to 0.6 V vs SCE.

The kinetics of an electrode are related to its reversibility, with faster kinetics associated with a more reversible system. However, reversible or irreversible behavior depends on the voltage scan rate; higher scan rates will encourage greater electrochemical irreversibility. The difference between a reversible process and an irreversible one is evaluated from the potential separation between the peaks in a cyclic voltammogram: Δ*E* = anodic *E* – cathodic *E*, where anodic *E* and cathodic *E* are the oxidation and reduction peaks, respectively.^43^

To study electrode kinetics, we compared the cyclic voltammogram behavior of pyrolytic vs graphitic carbon electrodes. In Figure 6a, we observe that Ohm’s law dominates the electrochemical response associated with pyrolytic carbon electrodes; in this case, the electrode behaves as if the reaction were irreversible (high Δ*E*), and it is not possible to clearly identify the peaks of reduction and oxidation of the redox couple. On the other hand, the graphitic carbon electrode (SU-8 + MWCNT and Ni-diffusion, see Figure 6b) exhibits a voltammogram with well-defined and symmetrical anodic and cathodic peaks, indicating a clear reversible process even at increased scanning rates. These results infer very rapid electron transfer kinetics between the graphitic carbon electrode and the electrolyte. We also verified that the maximum current density varies linearly with the square root of the scanning speed (*I*_p_ ∝ √ν), again characteristic of a reversible response (Figure S11).

**Figure 6:**
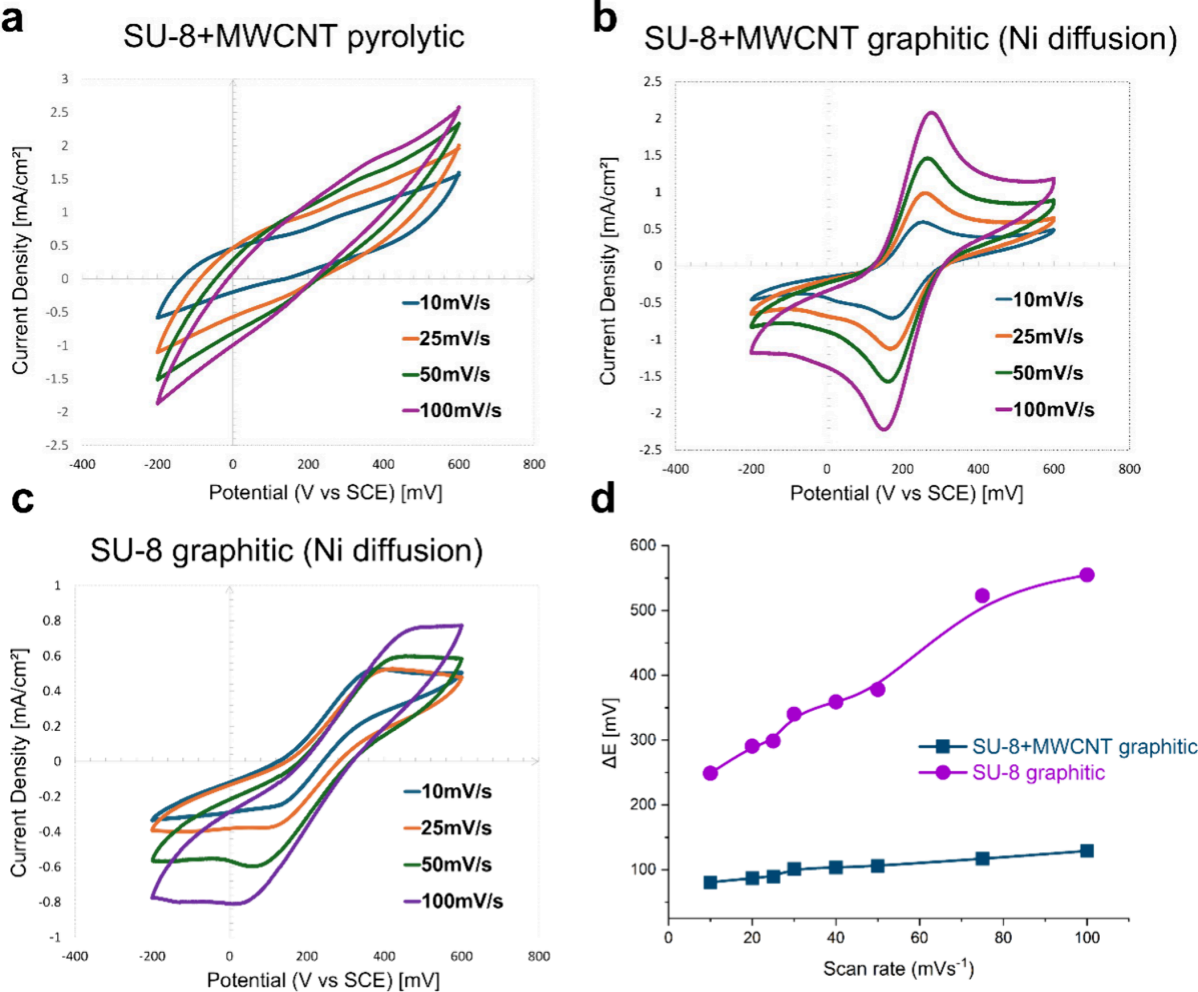
Cyclic voltammetric response of ferrocyanide (10 mM) with (a) SU-8+MWCNT pyrolytic electrode, (b) SU-8+MWCNT graphitic (Ni-based diffusion synthesis) electrode, and (c) SU-8 graphitic electrode (Ni-based diffusion synthesis), at variable scan rates. (d) Δ*E* vs. ν of SU-8+MWCNT and SU-8 graphitic electrodes (both with Ni diffusion). SU-8+MWCNT suggests highly efficient electron transfer kinetics, reflected in the very shallow slope. In stark contrast, the SU-8 electrode exhibits a pronounced scan-rate dependence of Δ*E*, a hallmark of irreversible kinetics.

To evaluate electrochemical performance quantitatively, we calculate the electron transfer rate constant *k*° between the electrode surface and the redox couple, using the Nicholson equation for single-electron reactions:^44^

4 $$\Psi \left(\Delta E_{p}\right)={\left(\frac{D_{O}}{D_{R}}\right)}^{1/2}k^{{}^{\circ}}{\left(\frac{\pi nF\nu D_{O}}{RT}\right)}^{-1/2}$$

Where *D*_O_ = 7.26 × 10^–6^ cm^2^/s, the ferricyanide diffusion coefficient, *D*_R_ = 6.67 × 10^–6^ cm^2^/s, the ferrocyanide diffusion coefficient, *F* is the Faraday constant, *R* is the gas constant, *T* = 298 K, and *v* is the scanning rate.

The obtained graphitic carbon from SU-8+MWCNT and Ni diffusion achieves a heterogeneous electron transfer rate constant (*k*°) of 0.064 cm/s at 100 mV/s. In Table 2, some heterogeneous *k*° values for various carbon materials are presented; SU-8+MWCNT graphitic exhibits a higher *k*° than materials commonly used for electrochemical biosensing applications, such as glassy carbon and highly ordered materials like HOPG.

**Table 2:** *k*° Values for Different Carbon Materials (Aqueous [Fe­(CN)_6_]^3–/4–^)

material	*k*° (cm/s)	ref
Monolayer graphene	1.5 × 10^–4^	^45^
CNTs	8.34 × 10^–5^	^46^
Glassy carbon	5 × 10^–3^	^47^
HOPG (basal plane)	1 × 10^–7^ to 10^–3^	^48^
Graphitic carbon from SU-8+MWCNT and Ni diffusion	0.064	Current work
Graphitic carbon from SU-8 and Ni diffusion	1.16 × 10^–5^ cm/s	Current work
Graphitic carbon from PAN+CNT + mechanical treatment	0.032	^29^

We also characterized the electrochemical behavior of the starting material without carbon nanotubes. In Figure 6c we present the cyclic voltammogram of such an SU-8 graphitic electrode (with Ni diffusion), which exhibits irreversible electrochemical behavior. This is evident from the large peak separation (Δ*E*) observed. We compare cathodic–anodic peak separations (Δ*E*) at various scan rates for both SU-8 graphitic and SU-8+MWCNT graphitic electrodes (Figure 6d). At a scan rate of 10 mV/s, the SU-8+MWCNT electrode exhibits a Δ*E* of 80 mV, nearing the reversible limit, while the SU-8 electrode shows a significantly larger ΔE of 248.88 mV (Table S11). Even at higher scan rates (e.g., 100 mV/s), the SU-8+MWCNT electrode maintains quasi-reversible behavior with a Δ*E* of 129 mV, which is notably lower than the SU-8 electrode’s Δ*E* of 248.88 mV at a 10-fold slower scan rate. These results indicate that the SU-8 electrode has less efficient electron transfer kinetics, as evidenced by its larger Δ*E* values across all scan rates compared to the SU-8+MWCNT electrode. The minimal variation in Δ*E* for the latter suggests highly efficient electron transfer, reflected in its shallow slope in the Δ*E* vs ν relationship. In contrast, the SU-8 electrode demonstrates irreversible kinetics, with Δ*E* exceeding 200 mV and a pronounced scan-rate dependence.

Since for irreversible behavior (Δ*E* > 200 mV) Nicholson’s method is not directly applicable, *k*° for the SU-8 graphitic electrode was calculated using the Klingler and Kochi equation:^49^

5 $$k^{◦}=2.18{\left(\frac{\alpha nFD\nu }{RT}\right)}^{1/2}e^{-\left(\alpha^{2}F/\left(RT\right)\right)\Delta E_{p}}$$

SU-8 graphitic reaches *k*° of 1.16 × 10^–5^ cm/s at 100 mV/s, indicating poor kinetics compared to SU-8+MWCNT. This behavior aligns with electrical conductivity measurements, which exhibit 3 orders of magnitude difference between the two materials. The higher conductivity of the SU-8+MWCNT graphitic electrode enhances charge transfer efficiency, resulting in an improved electrochemical response.

### MWCNT Contribution

To evaluate the contribution of MWCNT in our synthesis, we removed them from the composite, fabricated electrospun fibers of 70 wt % SU-8 photoresist and repeated the whole process of Figure 2 and their Raman and HRTEM characterization is presented in Figure 7. Pyrolytic (step 3) and graphitic (step 6) nanofibers (after Ni-based diffusion synthesis) exhibit the Raman spectra displayed in Figure 7a. Pyrolytic SU-8 has two pronounced peaks, D at ∼1346 cm^–1^ and G peak at ∼1584 cm^–1^, with no presence of the 2D band; the average *I*_D_/*I*_G_ is 0.95. The graphitic SU-8 Raman spectrum presents a narrow, sharp, and high-intensity G band at 1583 cm^–1^. The D band is located at 1349 cm^–1^, and the 2D band at 2695 cm^–1^. The average *I*_D_/*I*_G_ ratio is 0.37, which indicates that, as in the composite with MWCNT (*I*_D_/*I*_G_ = 0.3), a highly graphitic material was obtained. In summary, MWCNT in composite improves the *I*_D_/*I*_G_ ratio by only 0.3–0.7%. Fibers without MWCNT analyzed by HRTEM also revealed multilayer graphitic domains parallel to the fiber axis (Figure 7b), as well as localized bucky onions, bamboo, and chain-like tubular structures (Figure 7c), and strips formed by well-defined graphitic layers stacked (Figure 7d), which have been identified as nanoribbons.^50^ Which are similar ordered lattices and nanostructures observed in the fibers with MWCNT discussed above (Figure 4b, Figure 5). This analysis suggests that the diffusion process of carbon atoms through the metal catalyst is the main factor determining graphitization.

**Figure 7:**
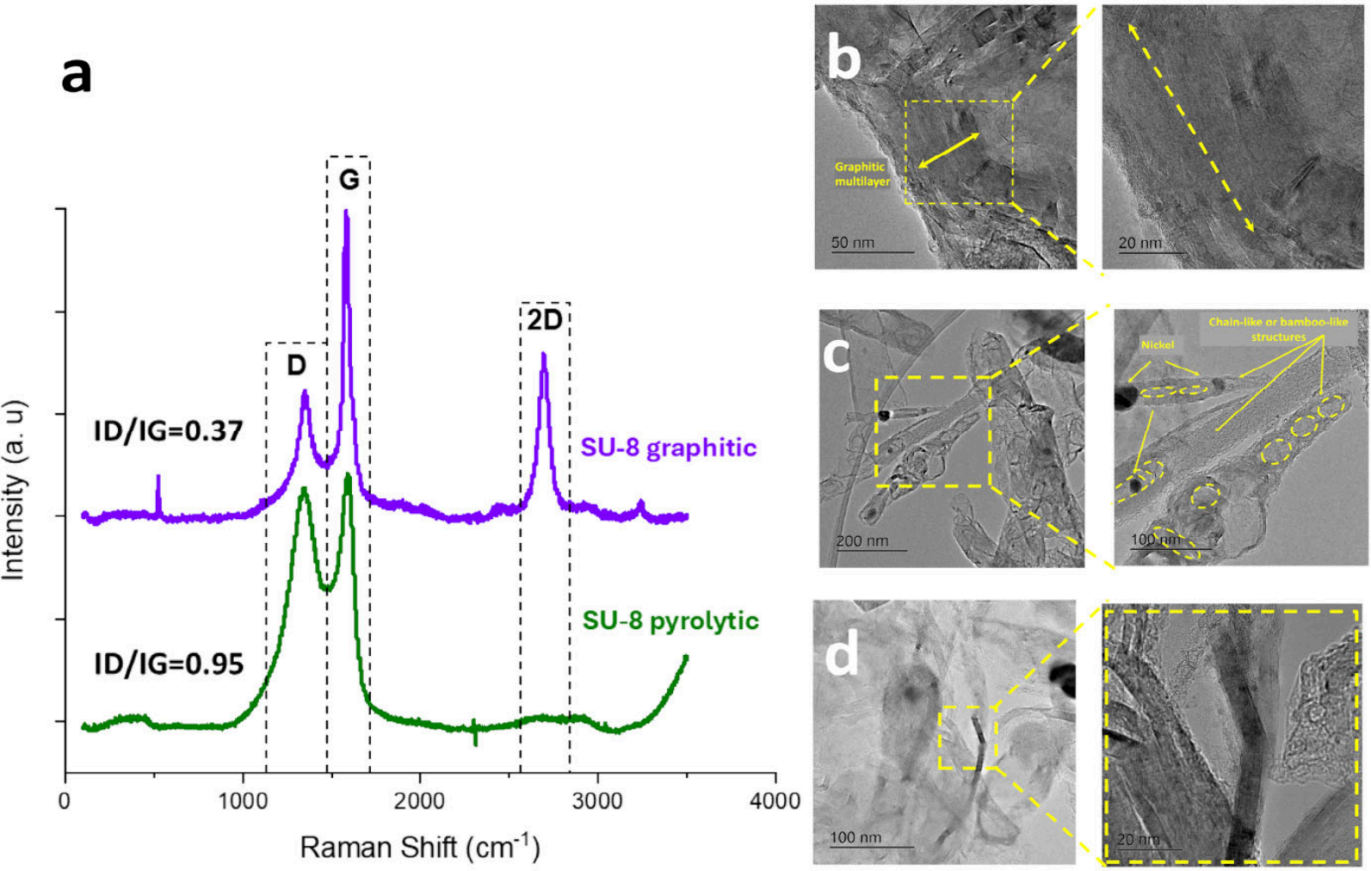
Structure of carbon derived from SU-8. (a) Raman spectra. The lower spectrum corresponds to SU-8 after pyrolysis, while the upper spectrum represents SU-8 after final synthesis. HRTEM of (b) graphitic ordering parallel to the fiber axis, (c) bamboo-like and chain-like tubular structures, and (d) graphitic layers stacked in the form of ribbons.

From our findings, the most notable effect of the MWCNT in the composite lies in the fiber’s morphological characteristics: SU-8-derived graphitic nanofibers exhibited significantly smaller diameters (∼115 nm) compared to SU-8+MWCNT composites (∼403 nm) under identical processing conditions, representing a 71% size reduction. This size disparity is visually evident in the SEM images (Figure 8a,b, 250 000× magnification) and quantitatively confirmed by the diameter histograms (Figure 8c). The structural difference correlates with a substantial conductivity contrast: SU-8 nanofibers demonstrated modest conductivity (CI_95%_ [1.97, 5.63] S/m), while MWCNT-incorporated exhibited a conductivity enhancement of 3 orders of magnitude (CI_95%_ [4638.1, 5625.9] S/m). To investigate more about this phenomenon, we indagate around the relationship between conductivity and porosity as well as percolation theory.

**Figure 8:**
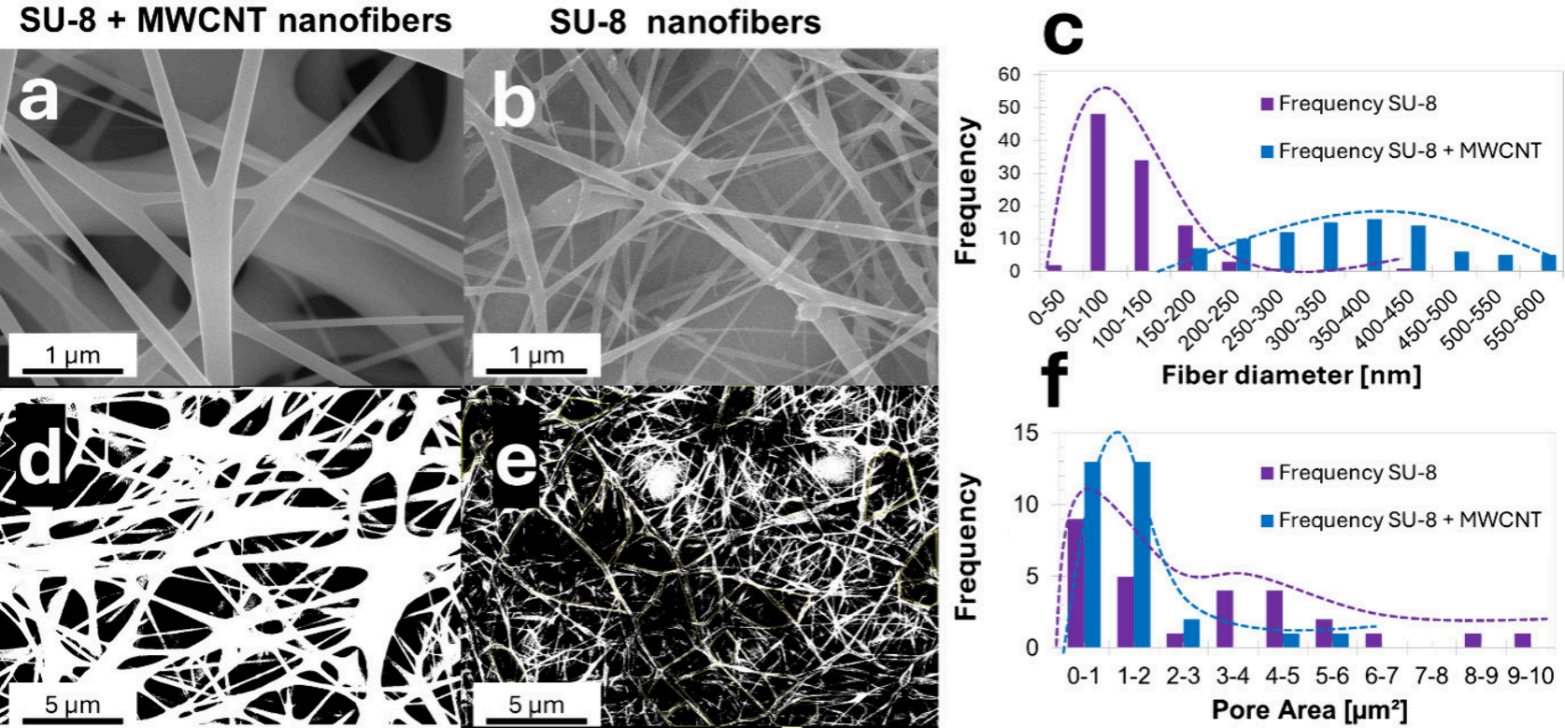
Fiber diameter and porosity of the nanofiber network after Ni-based synthesis. SEM micrograph at 250 000× magnification: (a) SU-8+MWCNT; (b) SU-8. (c) Comparison of fiber diameter histograms for the resulting carbons. SEM micrographs showing the porosity of the electrospun film at 5000× magnification: (d) SU-8+MWCNT; (e) SU-8. (f) Comparison of pore area histograms.

The total porosity is related to the pore size distribution, fiber diameter, and interconnectivity (the degree to which pores are connected to their neighboring pores), among other parameters^51,52^ which significantly influence resistivity. Montes stated that although there is no simple description to predict conductivity as a function of total porosity, it is intuitive that conductivity increases as porosity decreases.^53^ Reduced porosity is also related to an increase in packing density, and a more compact and conductive structure accelerates electron transfer,^54^ which is desirable in electrochemical applications.

Pore area measurements were conducted on electrospun films using SEM images at a magnification of 5000x. As shown in Figure 8d,e, SU-8+MWCNT composite exhibits a lower percentage of void space compared to the SU-8 nanofiber film. According to the pore area distribution histogram (Figure 8f), the SU-8+MWCNT composite exhibits an average pore area of 2.3 μm^2^, with most pores ranging between 0–2 μm^2^. In contrast, the carbon derived from SU-8 has a larger average pore area of 3.9 μm^2^. While most of its pores also fall within the 0–2 μm^2^ range, a considerable number of pores are observed in the 3–6 μm^2^ range, with some reaching sizes up to 10 μm^2^. In SU-8 graphitic carbon, the diameter of the nanofibers decreases, resulting in increased porosity. The other phenomenon related to this drastic electrical conductivity difference (with and without carbon nanotubes) is the percolation theory, our nanointerlaced carbon film works as a network of conductive fibers. According to this model, below a critical fraction of interconnected fibers, known as the percolation threshold (*p*_c_), no continuous conductive paths exist, and the material behaves as an insulator.^55^ Upon exceeding this threshold, a percolating network is established, and the conductivity σ­(*p*) increases sharply, following a power law described by the relation: σ­(*p*) ∼ (*p* – *p*_c_)^*t*^. The conductivity of the material (σ) increases as a power law once the volume of interconnected carbon fibers (*p*) exceeds a critical threshold (*p*_c_). How fast it increases is determined by the exponent (*t*).

This behavior is well-documented in composite materials, particularly those with nanofillers such as carbon nanotubes, where conductivity enhancements of up to 7 orders of magnitude have been reported just above the percolation threshold.^56–59^ Therefore, it is plausible to suggest that the variations in fiber morphology (e.g., diameter), film density, and pore size in our material are related to the interconnection of the carbon network, and the percolation phenomena described above can explain the remarkable increase in observed conductivity.

Although the effect of each parameter involved, such as fiber diameter, porosity, and interconnectivity in the fiber mat, adds complexity to the material behavior analysis, understanding them to be able to tune them will greatly impact the electrical and electrochemical microdevice’s performance.

## Conclusions

We have successfully developed a novel synthesis method for producing highly ordered nanointerlaced graphitic carbon from an SU-8 polymer incorporating a low concentration of MWCNTs. The process relies on Ni-based diffusion at 900 °C. By exploiting the photosensitive nature of the carbon precursor, this approach integrates the advantages of industrial photolithographic processing with enhanced material properties, achieving silicon-process compatibility while enabling precise control over graphitic microdevices without the need to apply any mechanical stress. The transformation from pyrolytic carbon (*I*_D_/*I*_G_ = 0.92, σ = CI_95%_ [615.0, 824.2] S/m) to graphitic structures (*I*_D_/*I*_G_ = 0.3, σ = CI_95%_ [4638.1, 5625.9] S/m) demonstrates the crucial role of catalytic diffusion in enhancing both structural order and electrical conductivity.

According to Oberlin, nongraphitizing carbons are characterized by an orientation range of less than 5–10 nm. Our Raman analysis reveals an increase in crystallite size (*L*_a_) from 5 nm in the pyrolytic material to 10–18 nm in the graphitic material. TEM measurements show an interplanar distance of 3.46–3.59 Å. These findings align with Ferrari’s description of graphite distorted by rotations, curvatures, and translations.

While the synthesis yields predominantly multilayer graphitic domains, it also generates localized nanostructures, including carbon nanocapsules, nano-onions, bucky onions, bamboo-like and chain-like tubular structures, and ribbons, resulting from the diffusion of carbon atoms. This complex hierarchical structure proves to be highly advantageous for electrochemical applications. The localized nanostructures inherently disrupt the continuous graphitic lattice, introducing grain boundaries and edge-plane defects throughout the nanointerlaced material. Consequently, while the parallel graphitic domains provide a highly crystalline framework that enhances bulk electrical conductivity, the structural discontinuities and edge defects serve as highly active electrocatalytic sites.

The demonstration that graphitic domains can be obtained at approximately 900 °C through thermal treatment and Ni catalysis of a photosensitive precursor opens new possibilities in materials science. The scalability of this synthesis is supported by several factors: (i) reliance on batch-compatible thermal processes, (ii) use of mature industrial technologies (electrospinning and thermal evaporation), (iii) elimination of graphitic layer transfer to a device substrate, and (iv) compatibility with parallel photolithography. Together, these features offer a clear pathway from laboratory-scale research to high-volume industrial production without modifying the underlying chemical synthesis.

Compared with solid electrodes, the nanointerlaced nature of our material offers distinct advantages for electrochemical sensing. Its inherent and tunable porosity significantly enhances the electroactive surface area, and the interconnected porous network facilitates ion/mass transport. Additionally, the 3D nanostructure provides superior bioreceptor immobilization stability compared with planar surfaces.

In our methodology, we employed a SU-8+MWCNT composite to assist in polymer chain prealignment during electrospinning and to enhance the material’s electrical conductivity. While the CNTs did not provide conclusive evidence of an improved graphitization degree (similar graphitization and carbon nanostructures were observed with and without MWCNTs), they exerted a significant impact on the electrical and electrochemical properties. Carbon derived from the SU-8+MWCNT composite exhibits a conductivity 3 orders of magnitude higher than that of SU-8 carbon. The electronic transfer rate constant (*k*°) for SU-8+MWCNT was 0.064 cm/s, 3 orders of magnitude greater than the 1.16 × 10^–5^ cm/s obtained for SU-8 material, confirming a direct correlation between high conductivity and efficient charge transfer at the electrode–electrolyte interface.

MWCNT incorporation resulted in a 70% increase in the polymer fiber diameter. Rheological measures ruled out viscosity as the primary factor governing this fiber thickening (Figure S12).

For SU-8 graphitic carbon (without MWCNT), the smaller fiber diameter may explain the reduction in conductivity. While porosity facilitates efficient transport kinetics, larger pore sizes can reduce the carbon structure density and fiber interconnectivity, leading to lower electrical conductivity compared to that of the SU-8+MWCNT composite under identical processing conditions.

This novel graphitic material demonstrates promise for next-generation electrochemical sensors due to its attractive charge-transfer characteristics, high conductivity, and tunable porosity. A thorough investigation of the role of carbon nanotubes in nanointerlaced material porosity is crucial for optimizing the balance between conductivity and electrochemical transport, which is essential to achieving maximum performance of this material. Its scalable fabrication and enhanced properties position it as a versatile candidate for point-of-care diagnostics, wearables, and microtechnology platforms, particularly in miniaturized, high-sensitivity, sensing devices.

## Supplementary Material





## Notes

Photolithography-defined micropatterning of nanointerlaced fibers, Raman spectroscopy data for SU-8+MWCNT graphitic and SU-8+MWCNT pyrolytic carbon, electrical conductivity measurements and statistical analysis, SEM-EDS, HRTEM-EDS, and cross-section SEM-EDS elemental analysis of nickel traces, electrochemical kinetic data, and precursor viscosity measurements (PDF)
Video S1 demonstrating the postexposure bake process of electrospun nanofibers (MP4)

## Abbreviations

CNT: carbon nanotube
MWCNT: multiwall carbon nanotube
PAN: polyacrylonitrile
GC: glassy carbon
CB: carbon black
CV: cyclic voltammetry
HRTEM: high-resolution transmission electron microscopy
SEM: scanning electron microscopy
CVD: chemical vapor deposition
RIE: reactive ion etching
