# Supplementary material for: Scalable Ni-Based Diffusion Synthesis of Highly Graphitic Nanointerlaced and Photopatternable Material with Fast Charge Transfer Kinetics
Source: Langmuir. 2026 May 21;42(24):17225–36. doi: 10.1021/acs.langmuir.5c06421 (PMC13296483; doi:10.1021/acs.langmuir.5c06421)
Supplement: Supplementary file 1 [file la5c06421_si_001.pdf]

Supporting information for:

Scalable Ni-based diffusion synthesis of highly graphitic nano-interlaced and photopatternable material with fast charge transfer kinetics

*Carina Chávez-Granados[a], Pedro Roquero[b], Oscar Pilloni[c], Margarita Rivera [d], Marc Madou[e] and Laura Oropeza-Ramos[a]**

[a] Facultad de Ingeniería, Universidad Nacional Autónoma de México, Ciudad Universitaria, Ciudad de México, 04510, México.

[b] Departamento de Ingeniería Química, Facultad de Química, Universidad Nacional Autónoma de México, Ciudad Universitaria, Ciudad de México, 04510, México.

[c] Instituto de Ingeniería, Universidad Nacional Autónoma de México, Ciudad Universitaria, Ciudad de México, 04510, México.

[d] Instituto de Física, Universidad Nacional Autónoma de México, Ciudad Universitaria, Ciudad de México, 04510, México.

[e] Tecnológico de Monterrey, School of Engineering and Sciences, Ave. Eugenio Garza Sada 2501 Sur, Monterrey, N.L., 64849, México.

1. Micropatterning of nano-interlaced fiber mat

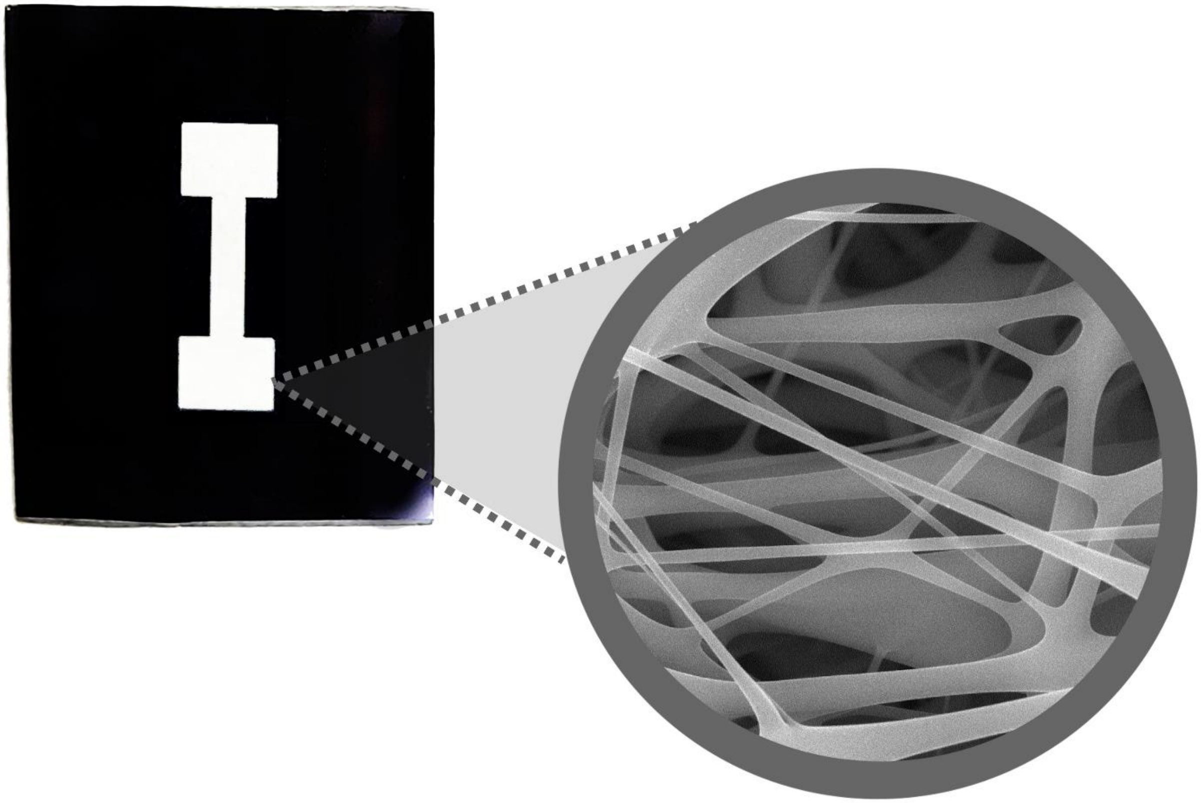

Figure S1. Photolithography-defined micropattern of SU-8+MWCNT nanofibers (280s exposure time) on silicon substrate.

Multimedia Files

Video S1 demonstrates the post-exposure bake (PEB) process of the electrospun SU-8/MWCNT nanofibers. This step is performed after the photolithographic exposure to define the micropatterns and ensures the cross-linking and structural stability of the composite material.

Video S1. Photolithography patterning of electrospun SU-8/MWCNT nanofibers avaible in: https://youtube.com/shorts/YiembzSMJ3w?si=QidNDzrQneVPtyLG

2. **Raman spectroscopy**

Table S1. Raman Spectroscopy measurements of SU-8+MWCNT graphitic (step 6-final)**.**

**Sample #**	**spectra**	**I_D [cps]**	**I_G [cps]**	**I_{2D} [cps]**	I_D/I_G
sample 1	1	35.268	87.225	36.298	0.4043336199
	2	23.149	71.789	32.519	0.3224588725
sample 2	1	14.428	48.095	25.578	0.299
	2	21.819	92.783	41.032	0.24896
sample 3	1	26.507	87.639	36.095	0.30245667
	2	22.038	91.8874	37.476	0.2398370179
sample 4	1	26.69355	84.36148	43.84875	0.3164
	2	30.21686	93.99786	44.86463	0.32146
sample 5	1	34.21054	110.3276	59.74012	0.31
	2	36.8148	115.3992	60.81067	0.319
sample 6	1	62.70689	195.777	98.76839	0.32029
	2	24.47978	88.48086	46.16337	0.27666
sample 7	1	52.25471	161.5839	87.60794	0.32339
	2	45.77501	152.5774	79.01138	0.3000117318
sample 8	1	52.9072	169.4657	90.7057	0.3122
	2	41.7736	155.3376	50.9239	0.268921
				**Average**	0.305336182
				**Standard Deviation**	0.03739409

Table S2. Raman Spectroscopy measurements of SU-8+MWCNT pyrolytic (step 3).

**Sample**	**I_D [cps]**	**I_G [cps]**	I_D/I_G
1	94.74822	105.5956	0.89727
2	97.5839	102.2242	0.9546
3	121.8109	129.7599	0.93874
4	89.6996	100.54	0.89217
5	81.2146	88.116	0.921678
6	41.7293	45.8783	0.90956
7	93.2631	101.062	0.92283

8	89.1675	96.9494	0.91973
		**Average**	0.9195722
		**Standard Deviation**	0.0205563

Bidirectional diffusion verification: Raman spectra were acquired following the methodology described in the Materials and Methods section to confirm bidirectional carbon diffusion. Measurements taken before Ni-etching (step 5) show an I_D/I_G ratio of 0.34 on the top surface, consistent with the $I_D/I_G = 0.3$ observed on the bottom layer after Ni removal (step 6).

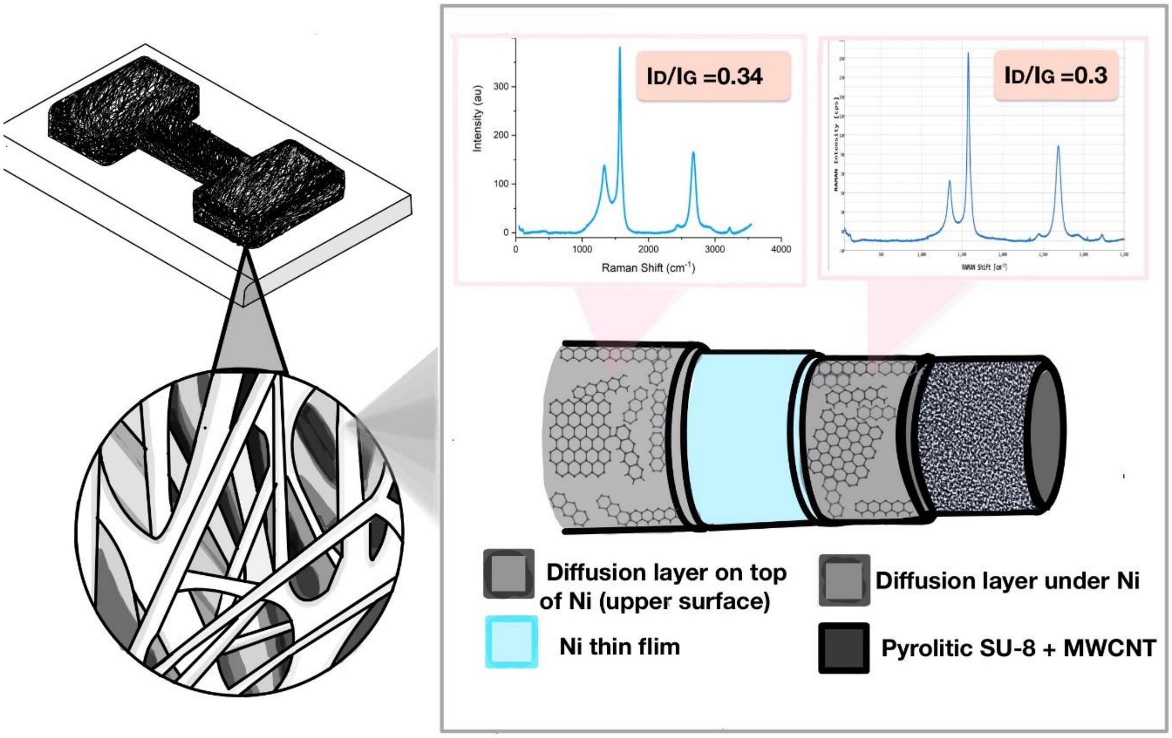

Figure S2. Schematic representation of bidirectional carbon diffusion in nano-interlaced SU-8+MWCNT graphitic films. The diagram illustrates the layered structure post-annealing, consisting of SU-8+MWCNT graphitic with a sacrificial nickel (Ni) thin film. Thermal treatment promotes interstitial carbon diffusion from the pyrolytic fibers into the Ni lattice, resulting in the simultaneous growth of graphitic layers on both the upper and lower surfaces of Ni. Raman spectra highlight the structural order of the material: the top diffusion layer (step 5, before Ni-etching) exhibits an $I_D/I_G = 0.34$, diffusion layer under Ni (step 6, after Ni-etching) shows $I_D/I_G = 0.3$.

3. **Conductivity measurements of pyrolytic (stage 3) and graphitic (stage 6-final) materials.**

Conductivity is measured at different stages of the fabrication process using the four-point probe method. The conductivity was measured across six independent samples for both the pyrolytic and graphitic steps. The data and statistical summary are presented in Tables S3-S5. As shown in Figure S3, there is no overlap between the CI of SU-8+MWCNT graphitic ([4638, 5626] S/m) and that of SU-8+MWCNT pyrolytic ([615, 824] S/m) and SU-8 graphitic (without MWCNT) ([1.97, 5.63] S/m). However, it must be considered that the nano-interlaced morphology limited the acquisition of I-V curves in two of the six original samples (n=4).

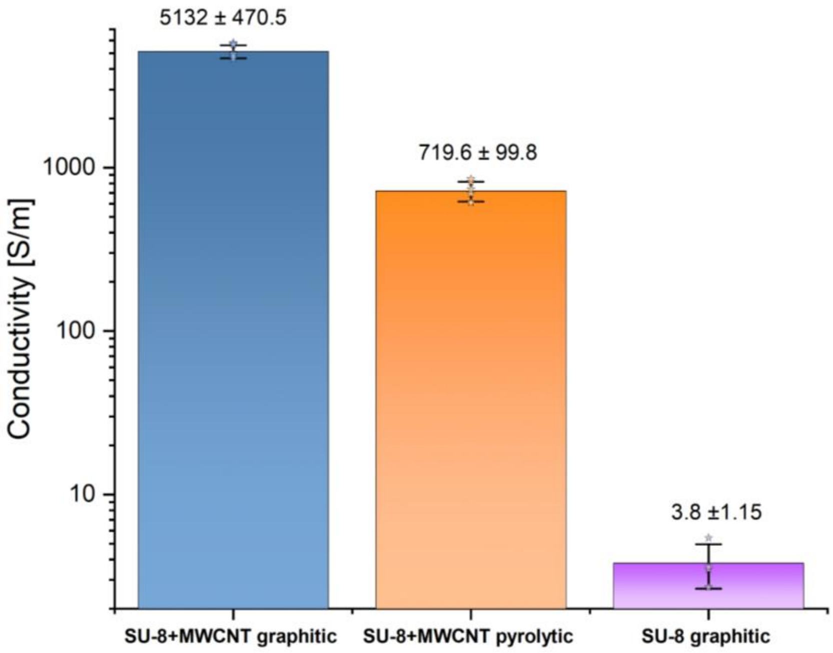

Figure S3. Comparative electrical conductivity. Bars represent the mean conductivity for SU-8+MWCNT graphitic, SU-8+MWCNT pyrolytic, and SU-8 graphitic samples. Individual measurements are shown as dots. Error bars indicate the standard deviation (SD). Numerical values above each bar correspond to the mean ± SD

Table S3. Experimental data and statistical metrics for electrical conductivity of the SU-8+MWCNT graphitic (step 6, with Ni diffusion).

**Sample**	**ΔV/I [Ω]**	**ρs [Ω/sq]**	**Conductivity, σ (S/m)**
Sample 1	5.4531	24.7157	5780.35
Sample 2	5.5775	25.2795	5651.1
Sample 3	5.7	25.8372	5000
Sample 4	5.5988	25.3757	4925.97
Sample 5	5.9492	26.9639	4636.5
Sample 6	5.7481	26.0526	4798
**Statistical metric**	**symbol**	**equation**	**value**
Mean	$\bar{x}$	∑σi/n	5132
Standard deviation	σ	∑(σi−x¯)2/(n−1)	470.5
Standard error	SEM	$\sigma/(n)^{1/2}$	192.1
Coeff. of variation	CV	(σ/x¯) ×100	9.17%
95%Conf. Interval	$CI_{95\%}$	$\bar{x} \pm (t_{\alpha/2,\, n-1} \cdot SEM)$	[4638.1, 5625.9] S/m

Table S4. Experimental data and statistical metrics for electrical conductivity of the SU-8+MWCNT pyrolytic (step 3, without Ni diffusion).

**Sample**	**ΔV/I [Ω]**	**ρs [Ω/sq]**	**conductivity, σ(S/m)**
Sample 1	17.87949	81.0363	617.0074
Sample 2	18.1061	82.06337	609.2852
Sample 3	18.803	85.222	690.2388
Sample 4	15.7636	71.4466	736.6562
Sample 5	15.0376	68.1558	815.1259
Sample 6	14.432	65.41146	849.3245

Statistical metric	symbol	equation	value
Mean	$\bar{x}$	$\sum\sigma i/n$	719.60
Standard deviation	σ	$\sum(\sigma i-\bar{x})2/(n-1)$	99.80
Standard error	SEM	$\sigma/(n)^{1/2}$	40.74
Coeff. of variation	CV	$(\sigma/\bar{x})\times 100$	13.86%
95%Conf. Interval	$CI_{95\%}$	$\bar{x}\pm(t_{\alpha/2,\,n-1}\cdot SEM)$	[615.0, 824.2] S/m

Table S5. Experimental data and statistical metrics for electrical conductivity of SU-8 graphitic (step 6, Ni diffusion without MWCNT).

**Sample**	**ΔV/I [Ω]**	**ρs [Ω/sq]**	**conductivity, σ(S/m)**
Sample 1	20372.8	92336.9	2.707478
Sample 2	31718.3	143759	3.478051
Sample 3	20419.1	92546.9	3.601778
Sample 4	20347.7	92223.3	5.421624
**Statistical metric**	**symbol**	**equation**	**value**
Mean	$\bar{x}$	$\sum\sigma i/n$	3.8
Standard deviation	σ	$\sum(\sigma i-\bar{x})2/(n-1)$	1.15
Standard error	SEM	$\sigma/(n)^{1/2}$	0.57
Coeff. of variation	CV	$(\sigma/\bar{x})\times 100$	30.24%
95%Conf. Interval	CI95%	$\bar{x}\pm(t_{\alpha/2,\,n-1}\cdot SEM)$	[1.97, 5.63] S/m

3.1 Math calculation of conductivity Sheet resistance ($\boldsymbol{\rho_s}$) and resistivity ($\boldsymbol{\rho}$) were calculated using the following equations:

$\rho_s = \frac{\Delta V}{I}\frac{\pi}{In2}$; Where $\boldsymbol{\rho_s}$ is sheet resistance and $\boldsymbol{\Delta V/I}$ is the reciprocal of the slope in the I-V curve.

And $\rho = \rho_s\, t;$ Where $\boldsymbol{\rho}$ is resistivity and $\boldsymbol{t}$ is the sample thickness.

Figure S4-S6 shows representative I-V curves and step-by-step calculations for the different carbons are provided to illustrate the methodology used. SU-8+MWCNT (Ni diffusion) shown in Figure S4, a sheet resistance ($\boldsymbol{\rho_s}$) of 24.715 and thickness ($t = 7\ \mu m$) was determined. Following the equations above, this yields a material conductivity (**σ**) of 5780.35 S/m.

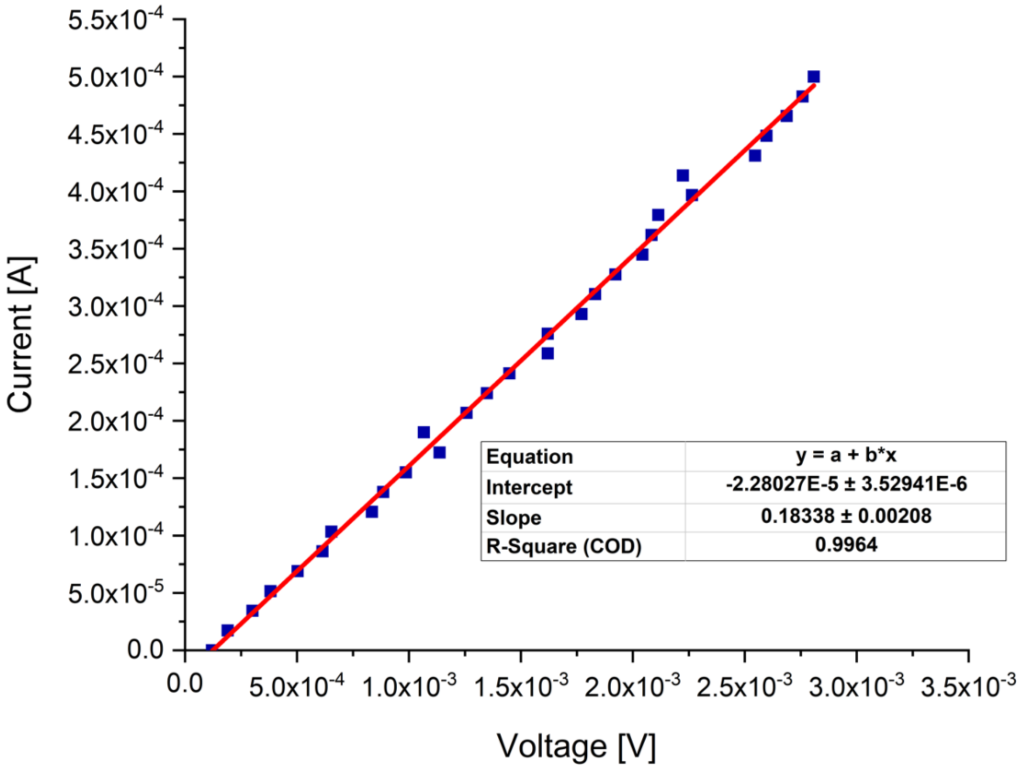

Figure S4. I-V plot of SU-8 + MWCNT after the Ni-based diffusion synthesis

Figure S5 shows an I-V curve for SU-8+MWCNT pyrolytic (step 3). A sheet resistance ($\boldsymbol{\rho_s}$) of 18.10610 and thickness ($t = 20\ \mu m$) was determined, corresponding to **σ** =609.28 S/m.

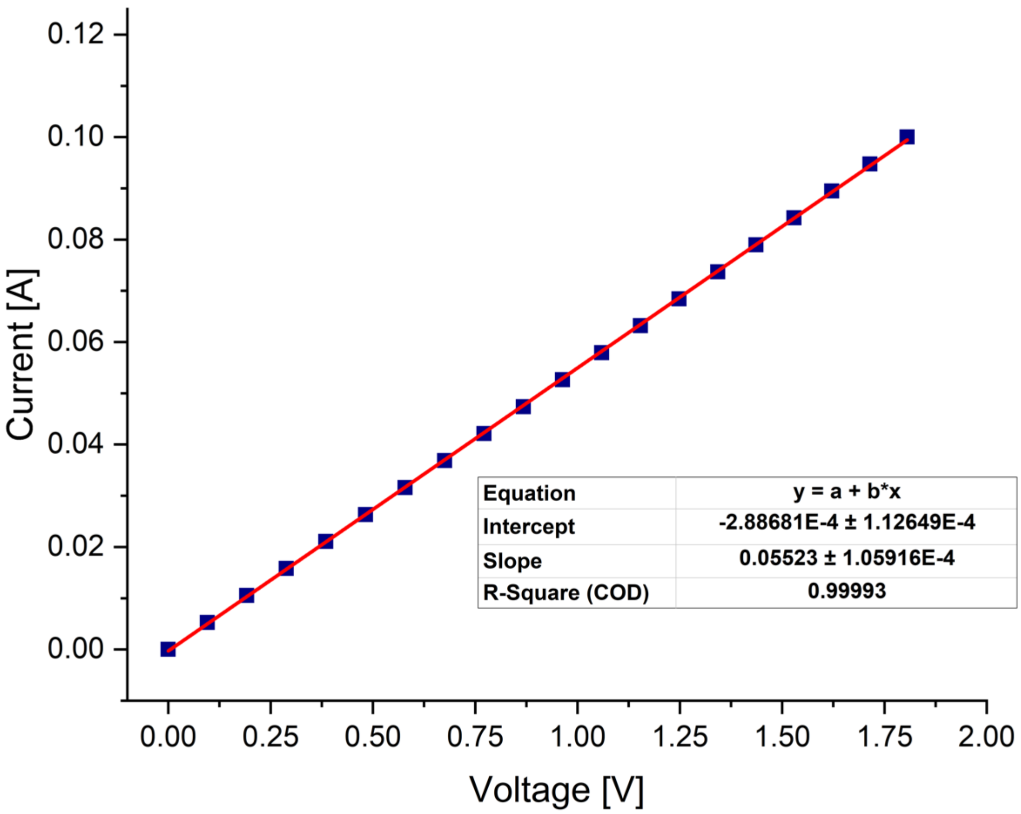

Figure S5. I-V curve of SU-8 + MWCNT after pyrolysis (step 3) without Ni diffusion synthesis.

Figure S6 presents the I-V curve for the graphitic material SU-8 (without MWCNTs and with Ni-based diffusion). A sheet resistance ($\boldsymbol{\rho_s}$) of 143758.681 was determined ($t = 2\ \mu m$), corresponding to a conductivity of 3.478 S/m.

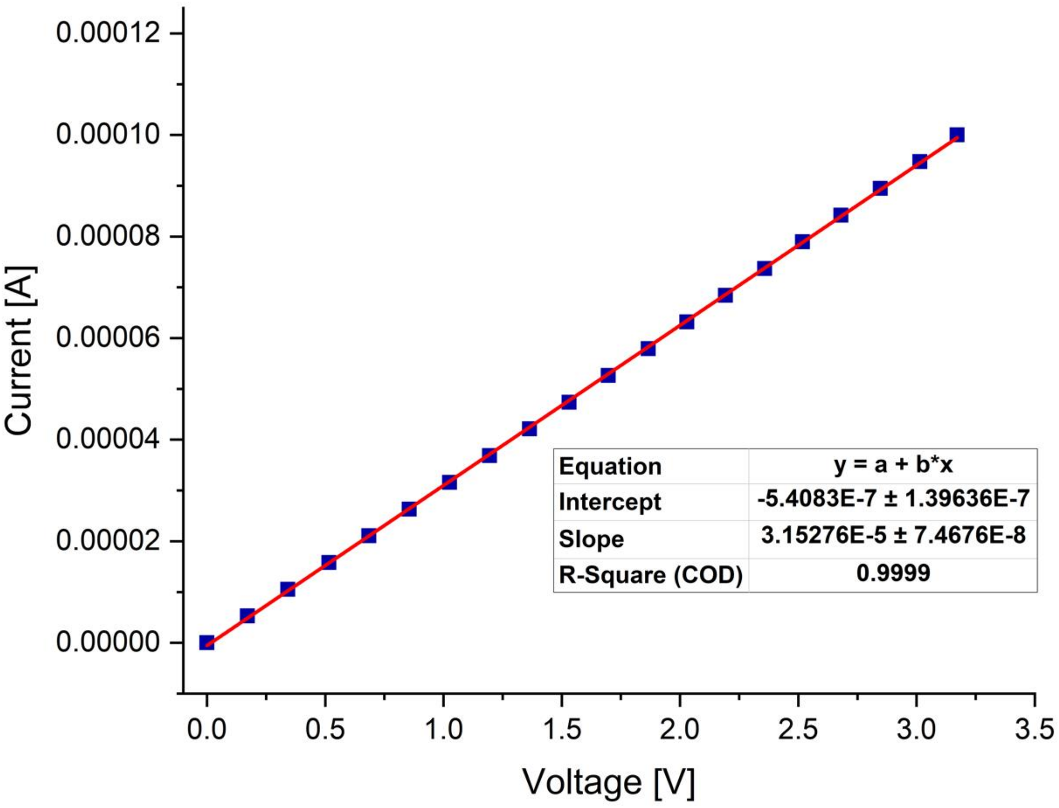

Figure S6. I-V curve of graphitic material derived from SU-8 (without MWCNT) after Ni-based diffusion synthesis.

3.2 Conductivity uniformity

To evaluate conductivity uniformity of the final material SU-8+MWCNT graphitic, n=3 independent samples were used, with 10 repeated measurements performed on each at random locations across the film (n_m=30 measurements).

Table S6. Statistical metrics for intra-sample and inter-sample conductivity of SU-8+MWCNT graphitic (6-step)

**Device**	**Mean [S/m]**	**Standard Deviation**	**95% Confidence Interval (CI)**
Sample 1	5141.38	396.62	[4857.66,5425.10]
Sample 2	5411.86	1125.58	[4606.67,6217.05]
Sample 3	5451.83	794.19	[4883.70,6019.95]

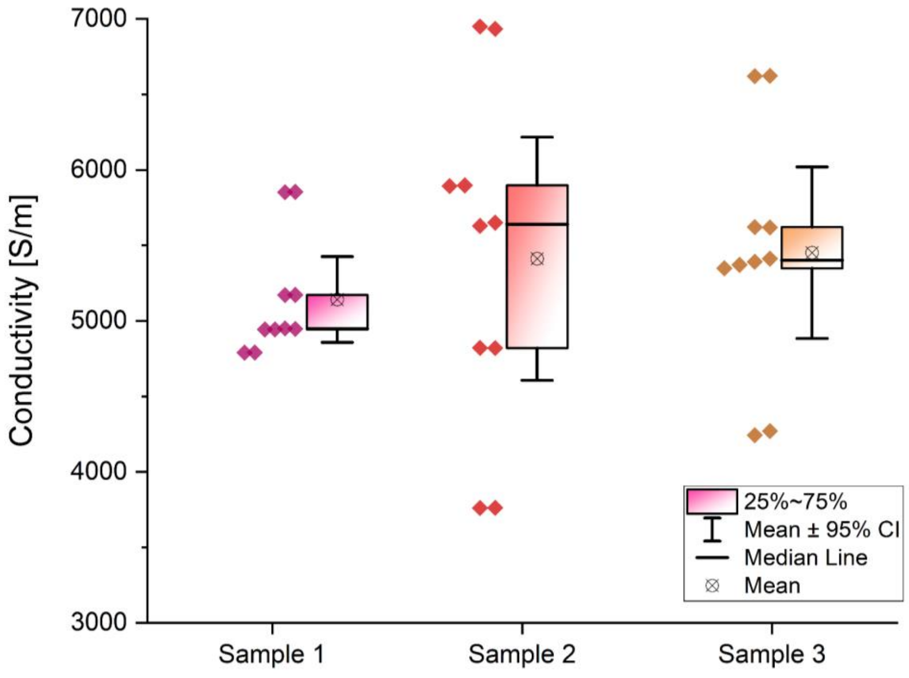

Figure S7. Box plot showing the conductivity distribution for independent samples.

To statistically test the hypothesis of uniformity, a One-Way ANOVA was applied. The null hypothesis H_0: $\boldsymbol{\mu}_1 = \boldsymbol{\mu}_2 = \boldsymbol{\mu}_3$ is not rejected since the p-value (0.6636) > $\boldsymbol{\alpha} = 0.05$, it indicates that the data are consistent with H_0, suggesting that the variation in conductivity is likely dominated by morphological arrangement of nano-interlaced fibers in the measurement region, rather than by systematic differences between synthesis.

4. Energy Dispersive Spectroscopy (EDS) of SU-8+MWCNT with Ni diffusion

4.1 SEM-EDS top layer analysis for large areas

The efficiency of Ni removal from SU-8+MWCNT graphitic (after FeCl3 etching) was quantitatively evaluated across three independent samples (from different batches, n=30 spectra). Measurements were collected from random 60x40 μm areas at 2000x magnification. Statistical evidence (Table S7) confirms that Ni was significantly reduced, with residual traces representing less than 0.5 At. % of the total atomic composition of the nano-interlaced carbon.

Table S7. Statistical summary of SEM-EDS large-area analysis of Ni (At. %) traces for the graphitic SU-8+MWCNT

Sample	Mean	SD	SE	CI 95 Lower	CI 95 Upper
Sample 1	0.375	0.07322	0.023154	0.322622	0.427378
Sample 2	0.093	0.100006	0.031625	0.02146	0.16454
Sample 3	0.316	0.047889	0.015144	0.281742	0.350258

Table S8. Data of SEM-EDS analysis (At. %) for the graphitic SU-8+MWCNT

Sample #	spectra #	C (% At)	O (% At)	Ni (% At)	Si (% At)	Cl (%At)
Sample 1	spectra 1	92.29	6.46	0.52	0.54	0.19
	spectra 2	92.11	6.6	0.4	0.67	0.21

	spectra 3	92.75	5.75	0.34	0.95	0.21
	spectra 4	91.79	6.59	0.4	0.94	0.28
	spectra 5	92.91	5.92	0.39	0.52	0.25
	spectra 6	91.74	6.07	0.33	1.69	0.16
	spectra 7	91.43	7.33	0.35	0.61	0.29
	spectra 8	92.43	6.2	0.23	0.89	0.24
	spectra 9	91.79	6.08	0.39	1.59	0.16
	spectra 10	92.25	6.62	0.4	0.49	0.24
Sample 2	spectra 1	91.54	6.75	0	0.89	0.82
	spectra 2	90.47	7.93	0.23	0.51	0.86
	spectra 3	90.56	7.16	0.19	1.28	0.82
	spectra 4	91.28	6.25	0.19	1.49	0.79
	spectra 5	90.44	8.01	0	0.71	0.84
	spectra 6	91.19	7.09	0.15	0.73	0.85
	spectra 7	90.61	7.65	0	1.43	0.85
	spectra 8	90.74	7.48	0.17	0.79	0.81
	spectra 9	90.94	6.63	0	1.65	0.78

	spectra 10	90.93	7.44	0	0.84	0.8
Sample 3	spectra 1	85.44	3.95	0.32	10.08	0.21
	spectra 2	85.06	4.02	0.29	10.44	0.19
	spectra 3	86.05	4.16	0.35	9.23	0.21
	spectra 4	85.63	5.01	0.38	9.4	0.23
	spectra 5	82.67	4.69	0.31	12.18	0.15
	spectra 6	84.89	4.76	0.32	9.84	0.19
	spectra 7	83.78	3.98	0.22	11.82	0.2
	spectra 8	85.66	5.01	0.31	8.77	0.25
	spectra 9	85.43	3.93	0.28	10.18	0.18
	spectra 10	84.47	4.31	0.38	10.63	0.21

4.2 HRTEM-EDS to distinguish between the graphitic matrix and localized Ni residues.

To determine the nature of the particles observed in HRTEM, a survey of 38 EDS spectra was conducted on the SU-8+MWCNT after Ni diffusion. The analysis, summarized in Table S9, distinguishes between the highly graphitic matrix where Ni remains at trace levels (0.15 ± 0.18 at. %), as illustrated in Figure S8. In contrast, localized regions (n = 5) identify residual Ni particles (22.8 ± 15.9 at. %), as shown in Figure S9.

Table S9. Statistical summary of HRTEM-EDS analysis (At. %) for the graphitic SU-8+MWCNT after Ni diffusion (n=38 spectra).

Region analysis	n	Carbon (At. %)	Oxygen (At. %)	Nickel (At. %)	Interpretation
Graphitic matrix	33	85.32 ± 11.69	14.53 ± 11.65	0.15 ± 0.18	High graphitic material: Ni content was detected only at trace levels
Catalytic Centers	5	69.26 ± 15.44	7.90 ± 4.14	22.8 ± 15.99	Localized encapsulated Ni nanoparticles.

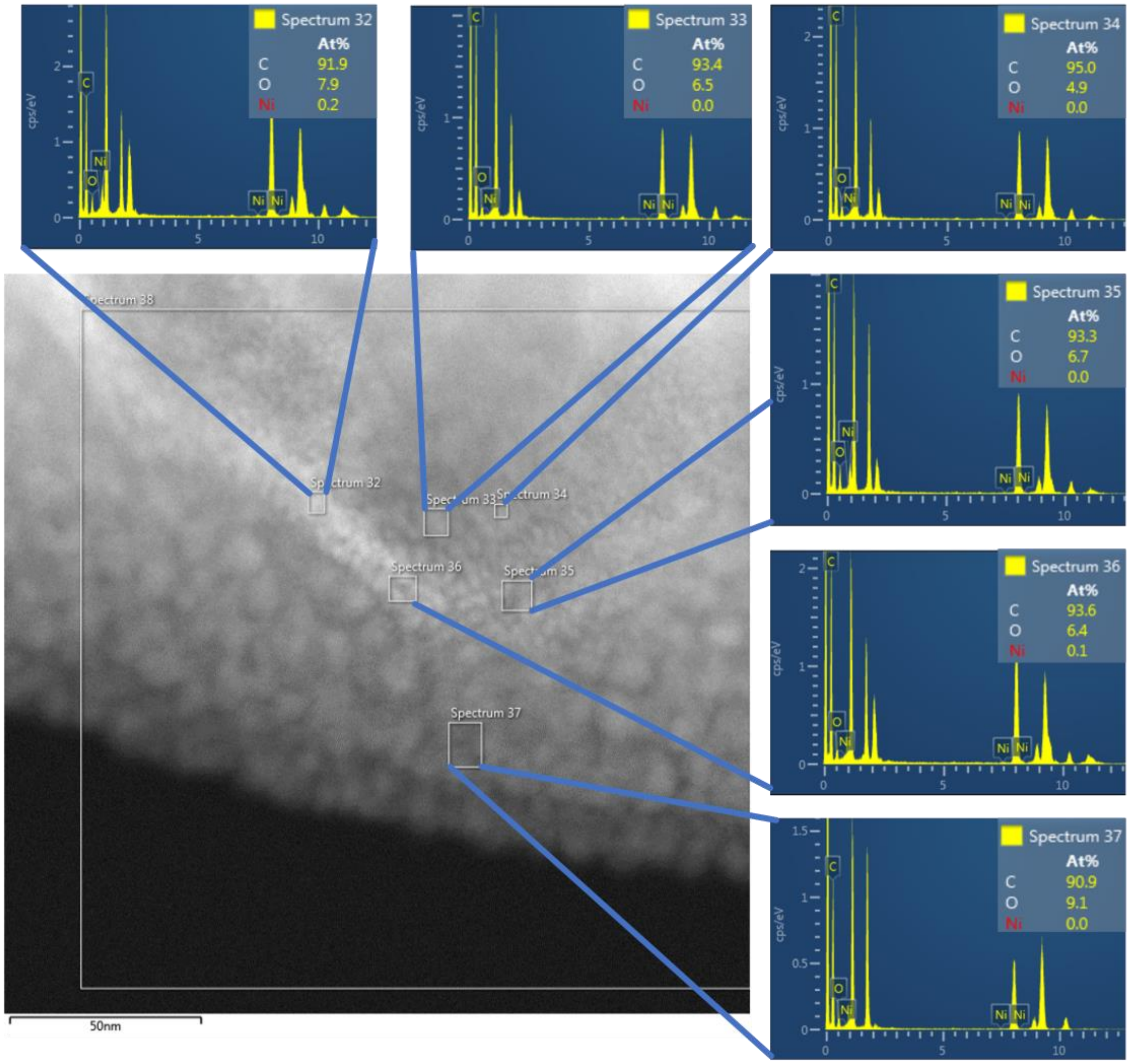

Figure S8. Representative EDS spectra of a graphitic nanofiber, Ni peaks remains at trace levels (0.15 ± 0.18 at. %)

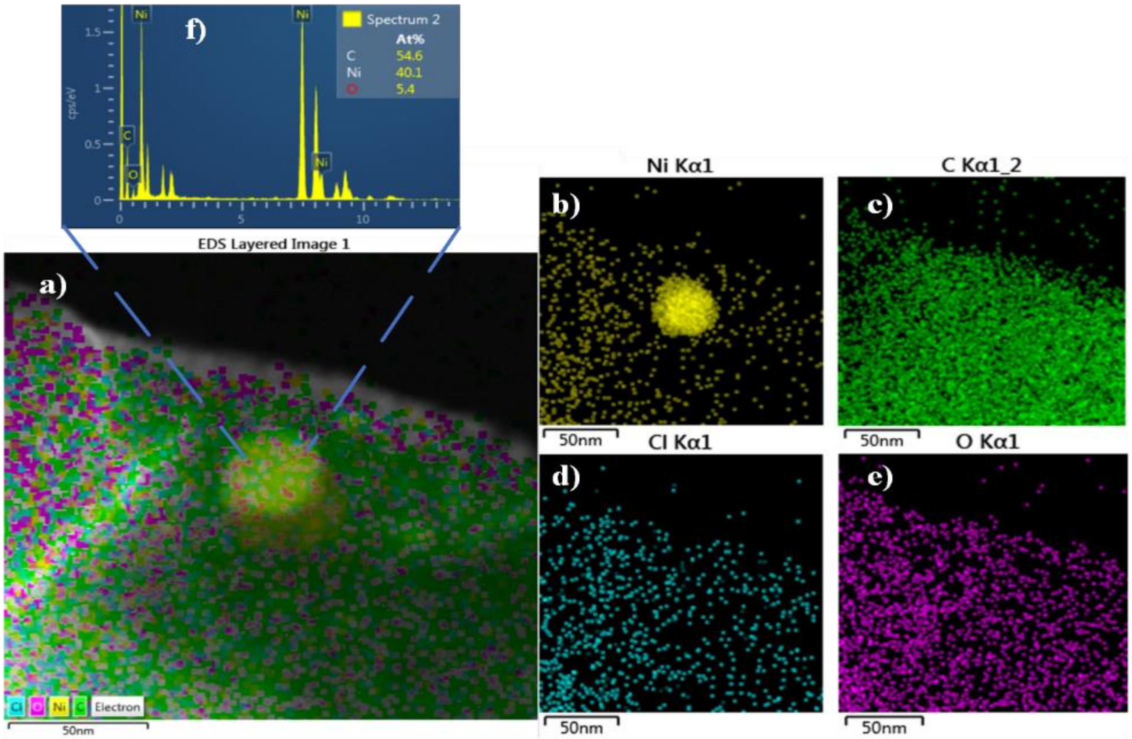

Figure S9. (a) Elemental analysis of a residual Ni nanoparticle (d=36 nm) is observed within the carbonaceous matrix. (b–e) Corresponding EDS elemental maps for nickel (Ni), carbon (C), chlorine (Cl), and oxygen (O), highlighting the spatial distribution of the elements. (f) EDS point spectrum acquired directly from the encapsulated Ni particle.

4.3 SEM-EDS of the cross-section at the final step of the synthesis

Elemental depth profiling was conducted on SU-8 graphitic mat cross-sections (after Ni etching step 6) across three independent specimens (n=9 spectra). The analysis confirmed that trace **Ni (0.27 ± 0.10 at. %)** is present throughout the film thickness, despite chemical etching (Table S10, Figure S10).

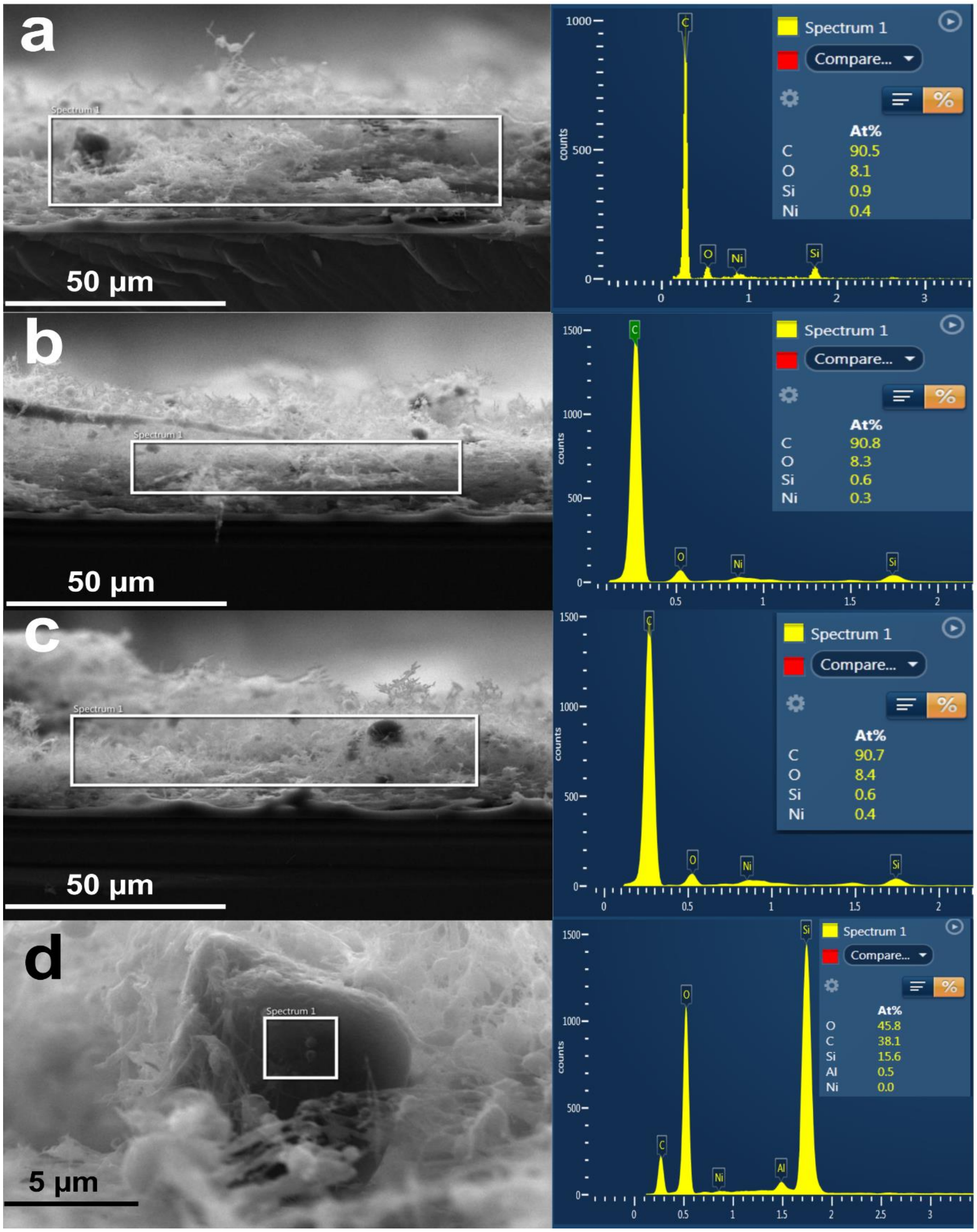

Figure S10. Cross-sectional SEM micrographs (a-c) of three independent samples, and elemental EDS mapping, Ni traces (low wt.%) detected within the internal regions. (d) High-magnification detail and EDS spectrum of a localized micro-particle identified as Silicon (Si); this feature likely corresponds to substrate debris from the cross-sectional cleaving process or a minor impurity, and it is not representative of the carbonaceous matrix.

Table S10. SEM-EDS elemental analysis (At. %) Cross- section of graphitic SU-8+MWCNT after Ni diffusion

Cross section Sample	Spectra #	C (% At)	O (% At)	Ni (% At)	Si (% At)
Sample 1	spectra 1	90.5	8.1	0.4	0.9
	spectra 2	93.1	6.2	0.2	0.5
	spectra 3	91.9	7.3	0.2	0.6
Sample 2	spectra 4	90.8	8.3	0.3	0.6
	spectra 5	90.3	9.2	0.1	0.4
	spectra 6	90.2	8.7	0.4	0.7
Sample 3	spectra 7	90.7	8.4	0.4	0.6
	spectra 8	90	9.2	0.3	0.5
	spectra 9	92.4	6.8	0.2	0.6
**Statics**	Average	91.1	8.0222	0.2777	0.6
	Standard deviation	1.09544	1.04854	0.1093	0.1414
	Standard error	0.3652	0.3495	0.03643	0.04714
	IC 95%	[90.26, 91.94]	[7.22, 8.83]	[0.19, 0.36]	[0.49, 0.71]

5. **Electrochemical Data and Randles-Ševčík equation**

Table S11. Comparison of peak separation (ΔE) for SU-8 and SU-8+MWCNT graphitic materials (Ni-based diffusion synthesis) at various scan rates.

**Scan rate [mVs^{-1}]**	**SU-8+MWCNT ΔE [mV]**	**SU-8 ΔE [mV]**
10	80.62	248.88
20	87	290.75
25	89.7	298.62
30	101.6	340.13
40	103.5	359
50	106	378
75	117	522.75
100	129	555

As shown in Figure S11, the peak current (I_p) demonstrates a strong linear relationship with the square root of the scan rate ($v^{1/2}$) (with a correlation coefficient $R^2 > 0.99$), where the current is limited by the diffusion of redox species from the bulk solution to the electrode surface. This behavior is related to the kinetic reversibility of the electrode.

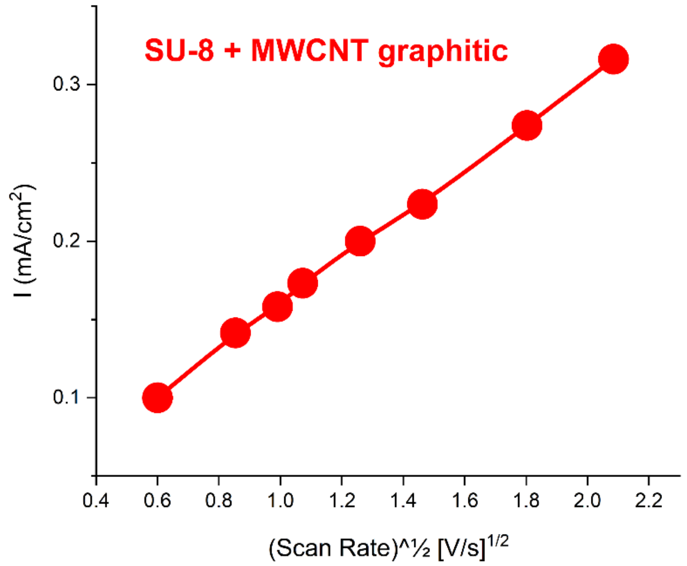

Figure S11. Randles-Ševčík curve of graphitic material from SU-8+MWCNT after Ni-based diffusion synthesis.

6. Viscosity measurements of the different precursors' preparation

Viscosity measurements of SU-8 and SU-8+MWCNT preparations for electrospinning were taken from 5 ml. This rheological test was conducted using a Discovery Hybrid Rheometer HR-3, and a cone-plate geometry. The shear rate range applied varied from 0.1 rad/s to 300 rad/s, with the temperature maintained at 25°C.

Figure S12 presents the viscosity versus shear rate for the SU-8 resin and SU-8+MWCNT composite. Both materials exhibit a slight shear-thinning behavior, where viscosity gently decreases as the shear rate increases. The viscosity of the pure SU-8 resin ranges from 0.56 to 0.48 Pa·s. In comparison, the composite's viscosity ranges from 0.46 to 0.42 Pa·s. The incorporation of MWCNTs results in a slight reduction in the viscosity of the material compared to the polymeric matrix.

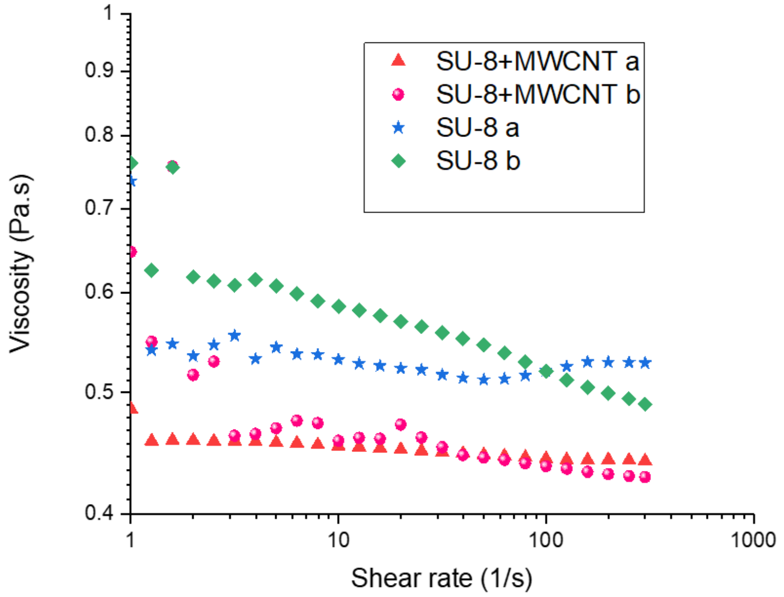

Figure S12. Viscosity versus shear rate curves for the SU-8 and the SU-8/MWCNT composite. Replicate measurements (labeled 'a' and 'b' in the legend) are shown for each sample. The two replicates for the composite (red triangles, pink circles) are highly consistent. While SU-8 replicates (blue stars, green diamonds) show some variability at low shear rates, both composite measurements are consistently lower in viscosity than the pure resin measurements across the entire range.
